# Crustal seismic velocity responds to a magmatic intrusion and seasonal loading in Iceland’s Northern Volcanic Zone

**DOI:** 10.1126/sciadv.aax6642

**Published:** 2019-11-27

**Authors:** C. Donaldson, T. Winder, C. Caudron, R. S. White

**Affiliations:** 1 Department of Earth Sciences, Bullard Laboratories, University of Cambridge, Cambridge, UK.; 2 Université Grenoble Alpes, University Savoie Mont Blanc, CNRS, IRD, IFSTTAR, ISTerre, 38000 Grenoble, France.

## Abstract

Seismic noise interferometry is an exciting technique for studying volcanoes, providing a continuous measurement of seismic velocity changes (*dv*/*v*), which are sensitive to magmatic processes that affect the surrounding crust. However, understanding the exact mechanisms causing changes in *dv*/*v* is often difficult. We present *dv*/*v* measurements over 10 years in central Iceland, measured using single-station cross-component correlation functions from 51 instruments across a range of frequency bands. We observe a linear correlation between changes in *dv*/*v* and volumetric strain at stations in regions of both compression and dilatation associated with the 2014 Bárðarbunga-Holuhraun dike intrusion. Furthermore, a clear seasonal cycle in *dv*/*v* is modeled as resulting from elastic and poroelastic responses to changing snow thickness, atmospheric pressure, and groundwater level. This study comprehensively explains variations in *dv*/*v* arising from diverse crustal stresses and highlights the importance of deformation modeling when interpreting *dv*/*v*, with implications for volcano and environmental monitoring worldwide.

## Teaser

Seismic velocity of the shallow crust changes after an intrusion of magma and seasonally due to environmental effects in Iceland.

## INTRODUCTION

Ambient noise interferometry is a promising tool for studying volcanic environments because relative seismic velocity changes (*dv*/*v*) of the crust are sensitive to stress and strain changes (*1*). Cross-correlation of the ambient noise wavefield allows continuous measurement of *dv*/*v* across large areas. Furthermore, by measuring *dv*/*v* in different frequency bands, it is possible to detect and distinguish changes across a range of depths, including those at which geodetic methods may not be sensitive (*2*). Magma pressurization at volcanoes (*3*), magmatic intrusions (*4*), changes in gas content (*5*), and precursors to volcanic eruptions (*6*) have so far been detected with *dv*/*v* measurements.

**Fig. 1:**
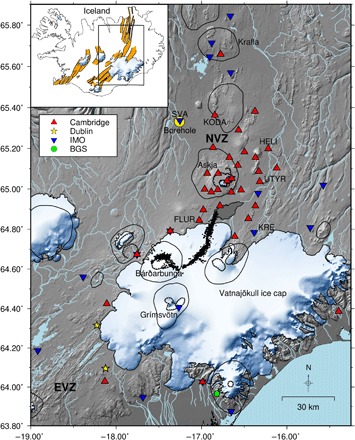
Station map. Seismic stations used in this study: University of Cambridge stations are shown as red triangles, University College Dublin stations as yellow stars, IMO stations as inverted blue triangles, and the British Geological Survey (BGS) station as a green circle. Borehole B5704 is shown by the yellow circle. Central volcanoes are delineated, with their calderas shown by ticked lines; earthquakes associated with the Bárðarbunga-Holuhraun dike intrusion are shown as black dots; and the erupted lava is in dark gray. Stations discussed throughout the text and the Northern Volcanic Zone (NVZ) and Eastern Volcanic Zone (EVZ) are labeled. Inset: rift segments are shown in orange.

There are 32 active volcanoes in Iceland; we measure *dv/v* using a network of seismometers across central Iceland, where the active volcanoes Grímsvötn, Bárðarbunga, Askja, and Krafla are located (Fig. 1), with particularly dense coverage in the Northern Volcanic Zone. In August 2014, magma intruded ∼50 km northward from Bárðarbunga volcano over a 2-week period before erupting in the Holuhraun lava field from August 2014 to February 2015 (*7*). The dike intrusion was delineated by over 30,000 microearthquakes at depth (*8*) and up to 4.5 m of opening, accommodated by graben formation, at the surface (*9*). Over the course of the eruption, 1.44 km^3^ of lava was erupted (*10*), producing volcanic tremor (*11*), and the ice-covered Bárðarbunga caldera subsided by more than 60 m (*12*). It became the largest eruption in Iceland in 230 years and provides an excellent opportunity to test the sensitivity of *dv*/*v* measurements to a major volcano deformation event. We model the strain caused by the dike intrusion and show that there is a linear relationship between volumetric strain and *dv*/*v* measured at individual stations, across both compressive and dilatational regions of the highly heterogeneous induced strain field. This highlights the importance of modeling when interpreting *dv*/*v*, particularly in volcanic environments where deformation associated with magma movements can produce complex strain fields.

Superimposed on the dike-induced signal, there is also a clear seasonal cycle in *dv*/*v* observed across a wide range of frequency bands (0.1–16 Hz). For *dv*/*v* to be implemented successfully as a volcano-monitoring tool in Iceland, this significant seasonal signal needs to be understood and accounted for. Moreover, it represents a second colocated natural forcing (and combination of stress changes) to which we can analyze the response of *dv*/*v*, with the prospect of improving our understanding of the factors controlling *dv*/*v* changes in the Icelandic crust. Several studies elsewhere in the world report seasonal variations in *dv*/*v* linked to changes in groundwater level (GWL) (*13*), rainfall (*14*), temperature (*15*), snow thickness (*14*, *16*, *17*), frost (*18*), and atmospheric pressure (*19*). As changes in these interlinked factors often occur at the same time, it can be difficult to extract the exact mechanism causing changes in *dv*/*v*. For example, positive correlations between snow thickness and *dv*/*v* have been observed in Japan in a 0.1–0.9 Hz band (*14*), at Mt. St. Helens for 1–5 Hz and 5–10 Hz bands (*16*), and at Mt. Etna for a 0.1–0.3 Hz band (*17*). These correlations could be caused entirely by increased snow load at the surface, stressing the underlying crust and causing microcracks to close. However, snow cover could also impede water infiltration into the ground, causing GWL to drop and, consequently, a decrease in pore pressure, which would also be expected to lead to a seismic velocity increase.

Elastic loading (from snow thickness and atmospheric pressure variations) is interpreted to be the primary cause of seasonal vertical displacements of the Icelandic crust measured by continuous Global Positioning System (GPS) stations in our study area (*20*). Seismicity rates have also been observed to vary seasonally in a geothermal area in this region (*21*), with more earthquakes occurring in the summer. However, as with *dv*/*v* changes, this correlation may be influenced both by elastic loading (increasing the confining stress in winter when the snow is thickest, thus suppressing seismicity) and by increased pore pressure, decreasing the effective confining stress in the summer. Analyzing *dv*/*v* measurements across a range of frequency bands that have different depth sensitivities enables us to separate out the effects of these potential causal mechanisms and to construct a model, combining elastic loading and pore pressure variations, which successfully explains the observed seasonal variation in *dv*/*v*. Thereby, we improve our understanding of the response of *dv*/*v* to a wide range of forcings, and we may also compare this to the magnitude of the signal from the dike intrusion, measured in the same region and with the same network of stations.

## RESULTS

We cross-correlate the waveforms measured by pairs of different components on the same instrument [single-station cross-components; (*22*)]. There are two main advantages to measuring *dv*/*v* from noise cross-correlation functions (NCFs) from single stations rather than pairs of stations. First, it is easier to interpret any spatial variations in *dv*/*v* as occurring in the vicinity of the station, within a volume related to the seismic wavelength (see the Supplementary Materials for further discussion), rather than scattered over a larger area around and between a pair of stations. Second, the signal-to-noise ratio of the coda of NCFs may be greater at rapidly attenuating high frequencies (providing information about *dv*/*v* changes at shallow depths), because the energy does not need to travel between a pair of stations as well as undergo additional scattering.

**Fig. 2:**
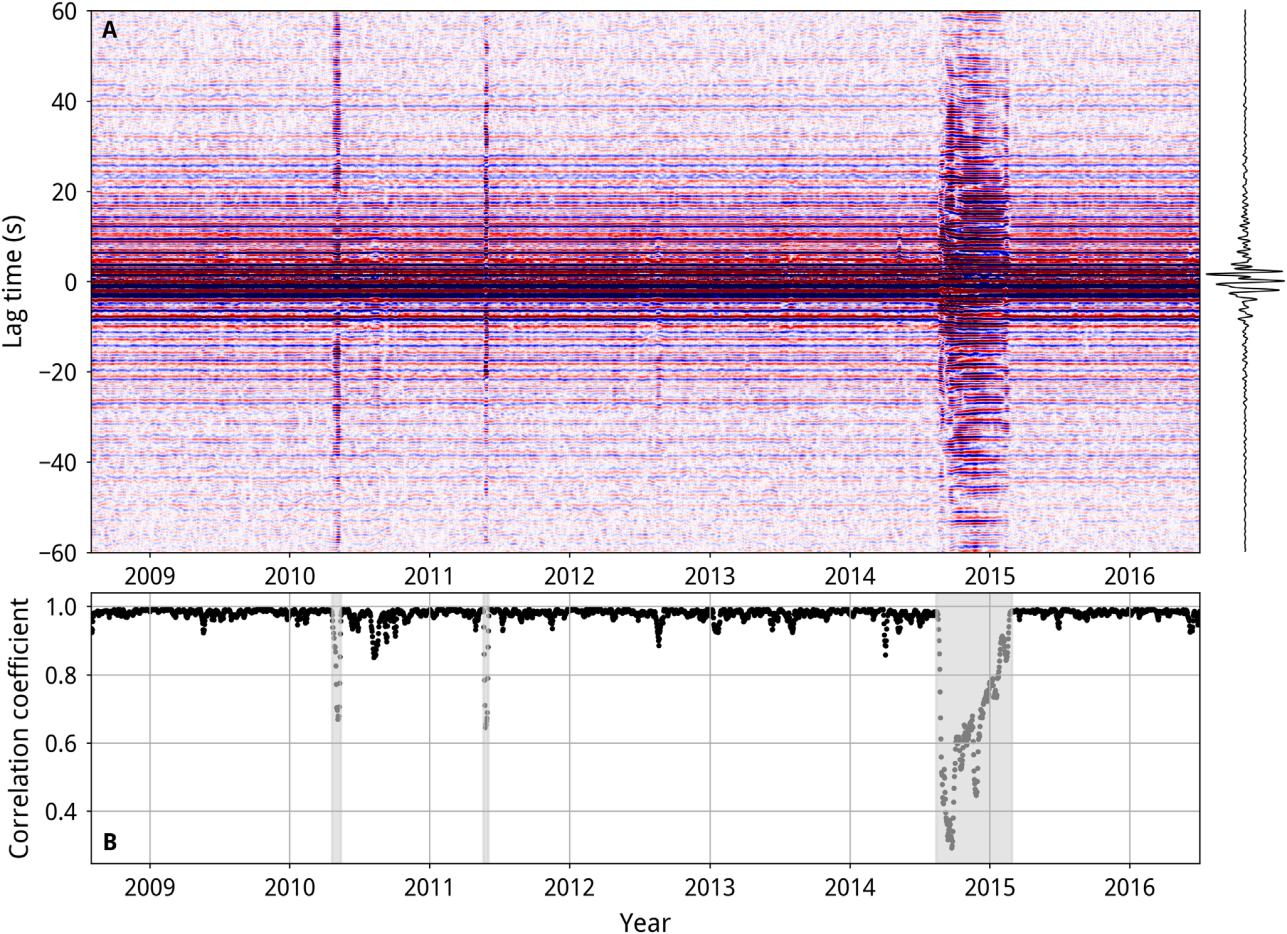
Example of single-station cross-component NCFs. (**A**) NCFs between the horizontal components at station UTYR in the frequency band 0.4–1.0 Hz, here stacked over 10-day windows. The reference function, a stack of all NCFs up to 15 August 2014, is shown to the right. (**B**) Correlation coefficient of the NCFs shown in (A) with the reference function, between ±120 s. The decreases in correlation coefficient in April 2010, May 2011, and August 2014 to February 2015 correspond to eruptions of the Eyjafjallajökull, Grimsvötn, and Bárðarbunga volcanoes, respectively. The durations of the eruptions are grayed out, indicating that *dv/v* measurements are rejected during these times.

An example of NCFs from station UTYR in the 0.4–1.0 Hz frequency band is shown in Fig. 2A. The NCFs are stable, and visual inspection suggests weak variations of the noise source over the study period, except during the eruptions of the Eyjafjallajökull, Grímsvötn, and Bárðarbunga volcanoes in 2010, 2011, and 2014−2015, respectively. Intense seismicity and volcanic tremor cause the NCFs to change visibly and, hence, the correlation coefficient with the reference function to decrease (Fig. 2B). We wish to isolate the signal produced by changes to the propagation medium, so we reject measurements of *dv*/*v* during these periods when there are significant changes in the noise source.

### Change in *dv*/*v* due to dike intrusion

**Fig. 3:**
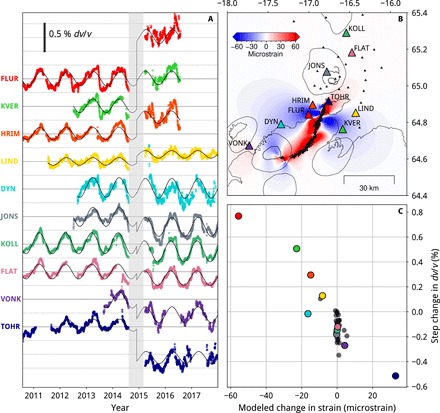
Strain modeling of dike intrusion and comparison with *dv/v* response at individual stations. (**A**) *dv/v* in the 0.4–1.0 Hz band for a selection of stations, 30-day stacks. The zero line is solid gray for each station; each horizontal dashed line is 0.25%. Fit of the time series according to Eq. 1 is shown in black. (**B**) Model of the volumetric strain field caused by the 2014 dike intrusion (details in Results). Negative strain (blue) is compression; positive strain (red) is dilatation. (**C**) Coefficient of the step in *dv/v* from before to after the rifting event [from (A)] against the modeled volumetric strain [from (B)] at each station; color codes are the same as in (A).

In Fig. 3A, we show *dv*/*v* measured with cross-component NCFs in the 0.4–1.0 Hz frequency band at a selection of stations. We use a stack of all NCFs before the 2014−2015 Bárðarbunga-Holuhraun eruption as a reference function for each single-station cross-component pair. Measurements at these frequencies have a high signal-to-noise ratio, are sensitive to the opening at the top of the dike (fig. S1), and have lateral sensitivity limited to a radius of ~5 km around each station (based on the first Fresnel zone and the wavelength; see the Supplementary Materials).

As well as high-amplitude annual variations, there are noticeable *dv*/*v* changes after the 2014−2015 Bárðarbunga-Holuhraun rifting event (the time period from the start of intrusion to the end of eruption is shaded in gray in Fig. 3A). For example, *dv*/*v* at station FLUR is ∼0.8% higher after the rifting event, whereas at TOHR, it is ∼0.5% lower. To estimate the change in *dv*/*v* at each station associated with the rifting event, we fit the *dv*/*v* time series with an ordinary least squares regression according to the following equation

$$\frac{dv}{v}=a\cdot \text{sin}(2\pi t)+b\cdot \text{cos}(2\pi t)+c\cdot S\cdot t+D$$ (1)

where *S* is a step function midway through the eruption, and the sine and cosine terms account for the annual cycle (discussed further below).

We compare these step changes in *dv*/*v* with the volumetric strain changes caused by the dike intrusion in August 2014. To calculate the dike-induced strain field, we use a model for the final dike opening obtained by inverting the surface displacements measured by GPS stations surrounding the dike, with the lateral extent of the dike constrained by the seismicity that tracked its propagation [as in (*23*)]. The same elastic half-space model and rheological parameters used to invert for the dike opening are used here to calculate the volumetric strain field; a slice at 1-km depth is shown in Fig. 3B. There are lobes of compressive (blue) strain on either side of the dike and a region of dilatation (red) to the north. In this simple dislocation model, all but the final 15 km of the dike are modeled to open to a minimum depth of 2 km below the surface, while the last segment (where the graben and eruptive fissures formed) opened to shallower depths. In reality, the dike opening probably tapered at its boundaries (*8*), removing some of the sharp changes seen in the modeled strain close to the dike. However, these apparent sharp changes occur closer to the dike than the stations used in this study, and so do not affect our conclusions.

In Fig. 3C, we combine these two independent observations and plot the coefficient of the step function in *dv*/*v* against the modeled volumetric strain change at each station. For the stations with an absolute modeled strain change of more than a few microstrain, i.e., those close to and strongly affected by the dike, there is a clear negative correlation. Station DYN is an outlier, showing a step decrease in *dv*/*v*, despite lying in a region where a positive volumetric strain change is modeled. This could be because of its proximity to the Bárðarbunga caldera (∼20 km away), which subsided >60 m during the eruption (*12*) and is not accounted for in this model of the strain changes caused by the dike intrusion.

There are small step decreases measured at many stations far from the dike, such as at KOLL and FLAT, where the modeled volumetric strain change is approximately 0. This results in a spread of the step coefficients in Fig. 3C between ∼0 and −0.3% at approximately 0 strain. This is likely to be because our simple model of a sinusoidal trend and step change is not always appropriate, for example, where there are additional long-term changes in *dv*/*v*. Long-term deformation in the Northern Volcanic Zone is controlled by the complex interplay of plate spreading, volcanic, and geothermal deformation at the Askja and Krafla volcanoes and glacial isostatic adjustment (*24*). Comparing *dv*/*v* with continuous GPS measurements would be an interesting future study.

**Fig. 4:**
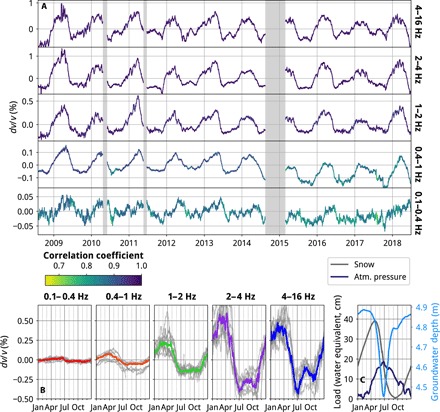
Continuous network-averaged *dv/v* measurements across five frequency bands. (**A**) *dv/v* measured from single-station cross-component NCFs with the stretching technique and averaged over the network. *dv/v* is measured by comparing 30-day stacks to a single reference function for the lowest two frequency bands, and between pairs of 5-day stacks offset by one day—with the earlier stack acting as a moving reference function—for the three higher frequency bands (see the Supplementary Materials for further details). The correlation coefficient is measured between windows of the stretched current NCF and the reference NCF; the time windows depend on the frequency band but are always within the NCF coda. (**B**) *dv/v* for each year is shown in gray, and the average is overlain in color. (**C**) Snow thickness (gray), atmospheric pressure (dark blue), and GWL (light blue) are averaged yearly over the same time period as the *dv/v* measurements. Elastic loading from snow and atmospheric pressure shown as water equivalent. GWL shown as depth below surface, i.e., a higher water level is plotted downward. Snow and atmospheric pressure are from the IMO’s meteorological model and GWL is modeled; see Results for details. Note that the *y* scales vary between the five panels of (A), but are consistent in the lowest panels (B and C).

The time series of *dv*/*v* are noisier after the 2014–2015 Bárðarbunga eruption (Fig. 3A), particularly for stations close to the eruption site. The correlation coefficients with the reference functions are also lower after the eruption (Fig. 4A), which is expected, given that the strain changes caused by this major deformation event likely alter the scattering paths of the noise wavefield.

### Yearly seasonal cycle in *dv*/*v*

To investigate the seasonal cycle in *dv*/*v*, we analyze results from five frequency bands between 0.1 and 16 Hz. For the 0.1–0.4 Hz and 0.4–1.0 Hz bands, we use a stack of all NCFs before the 2014−2015 Bárðarbunga-Holuhraun eruption as a reference function for each single-station cross-component pair, as outlined in the previous section. For the higher frequency bands (1–2 Hz, 2–4 Hz, and 4–16 Hz), initial measurements using this approach revealed large *dv*/*v* changes compared with the dominant period of the NCFs. We therefore follow the methodology of James *et al*. (*18*) and use 5-day-long moving reference functions, making daily *dv*/*v* measurements by comparing pairs of 5-day stacks offset by 1 day. Rayleigh wave sensitivity kernels (fig. S1) show that higher frequencies are sensitive to velocity changes concentrated at progressively shallower depths: The five bands we use, 0.1–0.4 Hz, 0.4–1.0 Hz, 1–2 Hz, 2–4 Hz, and 4–16 Hz, are sensitive over approximate depth ranges of 1 to 8 km, 0 to 3 km, 0 to 1.5 km, 0 to 400 m, and 0 to 100 m, respectively.

Figure 4 shows network-averaged relative seismic velocity variations (*dv*/*v*) observed during the period 2008−2018 in all five frequency bands between 0.1 and 16 Hz. There is a clear seasonal signal in *dv*/*v* across all frequencies. Annually, *dv*/*v* is high in the spring and low in the summer and fall, with peak-to-peak changes of ∼ 0.05 to 1.5%. The amplitude of the annual signal increases at higher frequencies, except for the 4–16 Hz band, where a slight reduction is observed. These results are averages of the single-station cross-component results across the network, measured using the stretching method. We find an excellent agreement if we instead calculate *dv*/*v* using the Moving-Window Cross-Spectral (MWCS) method (fig. S3) and with network-averaged measurements from NCFs calculated between pairs of stations (fig. S4), indicating the robustness of these results.

To compare the shape of the annual *dv*/*v* curve in each frequency band, we calculate yearly average time series over the entire time period (Fig. 4B). The timing of the peaks varies slightly each year, but in the 0.4–1 Hz, 1–2 Hz, 2–4 Hz, and 4–16 Hz bands, *dv*/*v* generally reaches a maximum around April then decreases over ~2.5 months to its minimum value. In the 0.4–1.0 Hz band, *dv*/*v* remains low between July and October, before increasing through the winter. For the 1–2 Hz, 2–4 Hz, and 4–16 Hz bands, *dv*/*v* instead generally starts to increase in July, often decreasing slightly in the fall before increasing again through the rest of the winter. In contrast, *dv*/*v* calculated in the 0.1–0.4 Hz frequency band reaches a maximum around June (around 6 weeks later than for the high frequencies), decreases over ~6 weeks, and then stays low before starting to increase in November. These patterns are generally consistent across the entire Northern Volcanic Zone, although in the south of the region—around the Vatnajökull ice cap, and close to the coast—trends are more varied (fig. S9).

To investigate the seasonal pattern in *dv*/*v*, we compare to weather data from the Icelandic Meteorological Office’s (IMO’s) reanalysis using the numerical weather prediction (NWP) model HARMONIE-AROME for the period August 2008 to June 2017 (*25*). This is downscaled from the ERA-Interim reanalysis, a global dataset of atmospheric parameters updated in real time. We average this model weather data across the Northern Volcanic Zone (in the region between stations KODA, FLUR, KRE, and HELI), where no weather stations are present.

Average annual time series of snow thickness and atmospheric pressure—both of which subject the crust to an elastic load—are displayed in Fig. 4C. The peak-to-peak annual amplitude change of atmospheric pressure is roughly half that of snow. First, we consider the average yearly trend in *dv*/*v* in the lowest frequency band (0.1–0.4 Hz). The shape of *dv*/*v* is similar to that of snow thickness, and the yearly peak in late June roughly corresponds to the peak in atmospheric pressure (the peak in snow thickness occurs at the beginning of May). This implies that *dv*/*v* in this lowest frequency band is primarily controlled by elastic loading of the crust. Furthermore, the timing and shape are also similar to the vertical GPS displacements observed in this region of Iceland (*20*), which have also been explained by the response to seasonal elastic loading of the crust.

To explain the earlier *dv*/*v* maximum in the higher frequency bands, we consider the annual variation in GWL, which results in significant variations in pore pressure at shallow depths. We calculate GWL by modeling the shallow crust as an aquifer with an exponential outflow (*26*)

$$\text{GWL}(t_{i})={\text{GWL}}_{0}-\sum_{n=0}^{i}\frac{p(t_{n})}{\phi }e^{\left(-a(t_{i}-t_{n})\right)}$$ (2)

where ϕ is the porosity, *a* is a decay constant, GWL_0_ is the asymptotic water level, and *p*(*t_n_*) is the water input at the surface. We consider snowmelt (assumed as any decrease in snow thickness) and rainfall (precipitation is assumed to be rain when there is no snow on the ground) as inputs of water at the surface. We derived values for *a* (0.06) and ϕ (0.24) by calibrating our model against GWL measured at borehole B5704, in the northwest of the study region (see fig. S9 and Fig. 1 for map). GWL is shown as depth below surface in Fig. 4C, with the groundwater depth taken to be the same as at borehole B5704.

The modeled GWL is also plotted in Fig. 4C, with a higher water level plotted downward. The GWL curve exhibits a much more spiky variation through the year, sharply increasing during the snowmelt around May, and peaking in June before quickly recovering most of the way to its minimum level through July and August. Upon initial inspection, the *dv*/*v* time series in the lower frequency bands (e.g., 0.4–1.0 Hz) appear to remain similar in shape to the snow thickness curve, while at higher frequencies (e.g., 4–16 Hz), the sharper drop and partial recovery between April and August show more resemblance to the GWL curve. However, *dv*/*v* does not precisely follow either snow thickness or GWL throughout the year in any of these highest four frequency bands. Instead, we suggest that a combination of the two factors, loading (snow and atmospheric pressure) and changing GWL, is necessary to explain the observed seasonal variations in *dv*/*v*.

## DISCUSSION

### Sensitivity of *dv*/*v* to dike intrusion

The excellent agreement between the modeled volumetric strain and *dv*/*v* provides a clear example of the sensitivity of *dv*/*v* to elastic strain changes in the crust caused by magma movements. Seismometers at some distance from the dike (e.g., FLUR is 7 km away) can easily detect the changes 6 months after the intrusion. The fact that interpretable signals can be measured in *dv*/*v* from just one station is exciting from a volcano monitoring perspective. However, both positive and negative changes in *dv*/*v* are observed as a consequence of the dike intrusion, due to the heterogeneous deformation field it produced. This highlights that modeling is crucial when interpreting *dv*/*v* variations in future work, particularly if only a few stations are available and in areas of laterally heterogeneous deformation, such as at volcanoes. Care needs to be taken when averaging over a network of stations, because some are likely to be in regions of compressive strain changes and others in dilatational regions, making the combined signal difficult to interpret.

A study in the eastern Izu peninsula of Japan observed changes in *dv*/*v* related to intrusions, but only decreases in *dv*/*v* were seen (there were no stations in the modeled compressional region of the strain field). There are several other examples of decreases in *dv*/*v* during volcanic events, such as directly preceding volcanic eruptions (*6*). However, a damage model, whereby brittle failure and the formation of new cracks cause a velocity decrease, was proposed to explain these observations (*27*). A similar nonlinear process is also needed to explain the drop in *dv*/*v* before the 2018 Kīlauea eruption; elastic deformation around an inflating magma reservoir is not sufficient to explain the observed drop in *dv*/*v* (*28*). In contrast, the increases in *dv*/*v* observed in this study, associated with compression from an intruding dike, show that the crust can behave elastically to first order.

The volumetric strain induced by the dike opening is, however, unlikely to be the only factor determining the behavior of *dv*/*v* over the course of the rifting episode. As described in the Results section, the step change in *dv*/*v* observed at station DYN is significantly lower than predicted by our model. Over the course of the intrusion and eruption, the Bárðarbunga caldera collapsed as magma flowed out into the dike and erupted at Holuhraun, resulting in up to 65-m subsidence of the ice surface (*12*). This deformation is not accounted for in our strain model, and Parks *et al*. (*29*) found that it led to significant stress (and therefore strain) changes extending tens of kilometers from the caldera rim, potentially accounting for the discrepancy at DYN. This may also affect the net volumetric strain change modeled at station VONK. Furthermore, we would expect a damage zone around the dyke, which might be expected to cause a decrease in *dv*/*v*, as described above. However, high-resolution relative relocations of microearthquakes associated with the intrusion suggest that this zone is narrower than ~300 m across (*8*). Given a maximum lateral sensitivity of ~5 km in this frequency band (see the Supplementary Materials), only station TOHR is likely to be sensitive to this damage zone. However, TOHR is also less than 1 km from the erupted lava, an additional elastic load, which we have also not considered [1.44 km^3^ of lava over an area of 84 km^2^ (*10*)]. This extra load would be expected to cause an increase in *dv*/*v* (in the same way as with increases in snow thickness and atmospheric pressure), which would act the opposite way to the potential decrease in *dv*/*v* caused by damage in the vicinity of the dyke.

We can further investigate these additional processes by studying changes in *dv*/*v* after the rifting event in different frequency bands, with varying lateral sensitivities. Unlike the method presented in Fig. 2, where we fit a step function to the continuous *dv*/*v* time series, we compare stacks of NCFs before and after the rifting event; the reader is referred to the Supplementary Materials for a full description. For the 0.4–1.0 Hz frequency band, the results shown in fig. S2D show excellent agreement with those in Fig. 3C, demonstrating the robustness of this observation. At 0.1–0.4 Hz and 1–2 Hz, we again observe a negative correlation between *dv*/*v* and strain. However, in the 0.1−0.4 Hz band, a lower amplitude *dv*/*v* change is measured for the same strain change, while in the 1−2 Hz band, a significantly higher *dv*/*v* is observed. In the 2−4 Hz band, the uncertainties in the *dv*/*v* measurements at each station are too large to interpret a trend. The correlation coefficients between the NCFs before and after the rifting event are very low, which suggests significant changes occurred to the scattering paths sensed by these shorter wavelengths over the course of the intrusion and eruption. The change in strain sensitivity with frequency band, and hence depth, probably reflects the degree to which the crust is cracked, and so, how it responds to stress changes. A very similar pattern is observed in the amplitude of the *dv*/*v* response to seasonal elastic loading with frequency (and depth), supporting this interpretation.

Figure S2A also shows that the *dv*/*v* change measured at DYN in the 0.1−0.4 Hz band (which has a large lateral sensitivity, like the 0.4−1.0 Hz band) also decreases after the rifting event, which again may reflect strain changes caused by the subsidence of the Bárðarbunga caldera. However, in contrast to the 0.4–1.0 Hz band result, *dv*/*v* at TOHR increases slightly at 0.1–0.4 Hz, perhaps due to the elastic loading from the erupted lava, as described above.

The velocity-stress sensitivity—the ratio of relative velocity change (*dv*/*v*) to applied stress perturbation—is a useful material property to measure, because it can indicate the compliance of the rock volume sampled (*1*). Taking a linear relationship (*R*^2^ = 0.80) between *dv*/*v* and the volumetric strain caused by the dike intrusion (Fig. 3C), we estimate a strain sensitivity of ~0.016 ± 0.001% per microstrain for the 0.4–1.0 Hz frequency band. Taking a value of 45 GPa for the Young’s modulus (*23*), this corresponds to a stress sensitivity of ~4 × 10^−9^/Pa. This is slightly below the range of values (5 × 10^−9^/Pa to 2 × 10^−6^/Pa) collated by Yamamura *et al*. (*30*). However, this is likely to be a minimum estimate because we can only measure *dv*/*v* after the eruption ends. By this time, ~6 months after the dike propagation, the crust may have relaxed to some extent, through viscoelastic or poroelastic processes. A significant recovery of ground deformation, modulated by poroelastic rebound, was observed after two large earthquakes in south Iceland over just a couple of months (*31*). Another factor that may contribute to underestimation of the stress sensitivity is that in modeling the dike-induced strain change at each station, we have used a point estimate of strain. In reality, *dv*/*v* is sensitive to a wider volume (maximum lateral sensitivity of approximately 5 km; see the Supplementary Materials), encompassing a strongly laterally varying strain field and also sampling both the very shallow, weak crust—which may not be able to sustain the strain changes—and deeper below the station, where the *dv*/*v* response is expected to be lower (as discussed previously).

### Modeling seasonal variation in *dv*/*v*

**Fig. 5:**
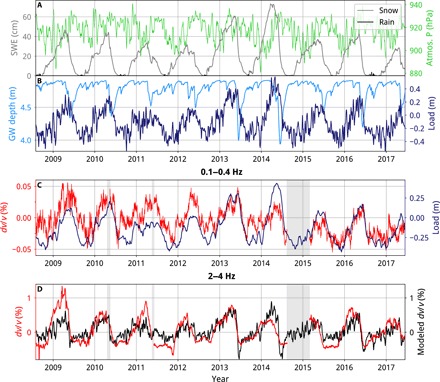
Model of seasonal variations in *dv/v* controlled by elastic loading and pore pressure changes. (**A**) Weather from IMO’s meteorological model averaged across the Northern Volcanic Zone (between stations KODA, FLUR, KRE, and HELI). Snow thickness as snow water equivalent (SWE) shown in gray. Rainfall, at the same scale, is in black. Atmospheric pressure is in green (1 hPa = 100 Pa). A 5-day rolling mean is applied to weather data for comparison with the stacked *dv/v*. (**B**) Modeled groundwater depth shown as depth below surface (see Discussion for details) shown in light blue. Modeled total load (combining snow, atmospheric pressure, and GWL; see Discussion for details) is shown in dark blue at the same scale. (**C**) Comparison of total load with *dv/v* measured in the 0.1−0.4 Hz band. A 30-day rolling mean is applied to the load because *dv/v* is stacked over 30 days in this frequency band. Gray bars show volcanic eruptions, as in Figs. 2 and 4. (**D**) Network-average of *dv/v* measured in the 2−4 Hz band (5-day stack, average of components NE, NZ, and EZ) is shown in red. Modeled *dv/v* is in black (see Discussion for details).

To examine the seasonal pattern of *dv*/*v* in more detail, we start by considering *dv*/*v* in the lowest frequency band: 0.1–0.4 Hz. Unlike the higher frequency bands, the maximum *dv*/*v* occurs around June, at approximately the same time as the maximum in atmospheric pressure (Fig. 4C), hinting that *dv*/*v* may be responding solely to a change in confining pressure due to a varying overlying load. We model the seasonal variations in load by combining snow thickness, atmospheric pressure, and GWL; the full time series of these variables are shown in Fig. 5. An important consideration is the time at which we define the snow load as having left the system. From the borehole data (fig. S10), it is clear that the water input during the snowmelt dominates the seasonal GWL changes, indicating that much of the melt water percolates into the crust, rather than escaping the system as surface runoff. On the basis of this observation, we make the simplifying assumption that all the water from both snowmelt and rainfall enters the crust. This means that the snow load will—initially—remain within the system, but in the form of water within the pore spaces of the shallow crust, instead of snow on top of it. This part of the elastic load is represented by the GWL, equivalent to the thickness of snow that has melted, but corrected for the porosity of the crust

$$\text{load}\;(\mathrm{Pa})=t_{\text{snow}(\text{SWE})}\cdot g\cdot \rho_{w}+P_{\text{atm}}+\frac{\text{GWL}\cdot g\cdot \rho_{w}}{\phi }$$ (3)

where *t* snow (SWE) is the thickness of snow on the surface as snow water equivalent. We estimate the porosity by fitting our GWL model to the measurements at borehole B5704 (Fig. 1), giving a value of 24%, which is reasonable for unaltered Icelandic lavas (*32*). There is a trade-off between the decay term (*a*) and porosity (ϕ) in Eq. 2, so our estimate of porosity is not well constrained. However, for the purpose of this analysis, the value of these parameters is not of great importance. Our model relies mainly on the shape of the GWL time series, which we are able to closely recreate (fig. S10), allowing us to calculate GWL for comparison with the network-averaged results.

In Fig. 5C, we compare network-averaged *dv*/*v* in the 0.1–0.4 Hz band with the total load; there is a remarkable positive correlation each year (Pearson’s correlation coefficient of 0.64). We therefore model *dv*/*v* as being proportional to load

$$\frac{dv}{v}=A\cdot \text{load}$$ (4)

resulting in a coefficient, *A*, of 0.07%/m.

However, as was explained in the Results section, a combination of loading and changing GWL (a proxy for pore pressure) appears necessary to explain the observed seasonal variations in *dv*/*v* in the higher frequency bands (particularly 2–4 Hz and 4–16 Hz). We focus on *dv*/*v* measured in the 2−4 Hz frequency band (Fig. 5D) because it is sensitive at shallow depths where pore pressure variations are closely tied to GWL (which is well constrained by the borehole data) and hence most appropriate for comparison to our calibrated GWL model. The time series of *dv*/*v* in the 4−16 Hz band is very similar to that in the 2−4 Hz band, so we suggest that the observations are also appropriate here. Later, we consider the *dv*/*v* variations in the intermediate frequency bands, corresponding to greater depths where pore pressure variations are less well constrained.

As was first seen in Fig. 4 (2–4 Hz and 4–16 Hz results), by studying Fig. 5 (A, B, and D), we again see that *dv*/*v* sharply decreases at the same time as the snow melts and GWL rises (around May and June), but it follows neither the GWL nor the snow thickness through the rest of the year. Generally, however, *dv*/*v* is positively correlated with the snow thickness and negatively correlated with the GWL, so we consider the effects of adding an elastic load (increasing the confining pressure) and a drop in GWL (a decrease in pore pressure) as the two main mechanisms causing *dv*/*v* to increase (*14*, *16*). We construct a linear model of the effect of GWL (as a proxy for pore pressure variations in the shallow crust) and loading on *dv*/*v* according to the following equation

$$\frac{dv}{v}=A\cdot \text{load}+B\cdot \text{GWL}$$ (5)

where the instantaneous effect on pore pressure can be estimated by multiplying the GWL by the water density, ρ_w_, and the acceleration due to gravity, *g*. We fit *dv*/*v* using Eq. 5 with an ordinary least squares regression; the modeled *dv*/*v* is shown in Fig. 5D. The resulting coefficients *A* and *B* are 1.1 and −1.3%/m, respectively, suggesting that the stress sensitivities of *dv*/*v* for the two mechanisms are of the same order (10^−6^/Pa) and of opposite sign. This is reasonable, given the linear relationship between effective stress σ_e_, normal stress, σ_N_, and pore pressure ϕ

$$\sigma_{e}=\sigma_{N}-\phi$$ (6)

It is important to realize, however, that this does not imply that the elastic loading and pore pressure effects of, say, a 10-mm rise in GWL will cancel each other out (*33*). For a porosity of 24%, a 10-mm rise in GWL corresponds to just 2.4 mm of water above the surface (or water load), so the effect of the pore pressure increase on *dv*/*v* will be about four times greater than the opposing effect from the increased elastic load. This is in agreement with several studies that consistently show a negative correlation between *dv*/*v* and rainfall (*14*, *34*, *35*) or GWL (*13*, *26*), suggesting that increasing pore pressure generally has a bigger effect than the increasing elastic load. Detailed explanations of the various effects of water input at the surface on pore pressure are given by Talwani *et al*. (*36*) and discussed in the Supplementary Materials; this would be a helpful basis for future studies where weather data and permeability structure are better known.

Teasing apart the mechanisms causing the seasonal change in *dv*/*v* in the higher frequency bands was initially difficult, given that several potential seasonal forcings occur simultaneously. However, our simple model, combining observations of environmental loads and GWL, successfully explains the observed annual changes in *dv*/*v*. Including both GWL and load improves the *R*^2^ value from models that only include one or the other (from 0.17 or 0.10 to 0.48), as well as improving the Akaike information criterion, which accounts for the trade-off between the goodness of fit and the simplicity (number of parameters) of the model. This reinforces the point that a combination of elastic loading and pore pressure changes is necessary to explain the seasonal changes in *dv*/*v* observed at high frequencies in this study. Groundwater behavior as measured at the borehole may not be representative of the region as a whole. However, in fig. S11, we show that our seasonal model (based on Eq. 5) can also reasonably fit *dv*/*v* measured at station SVA, which is 0.6 km from the borehole.

### Mechanisms of seasonal variation in *dv*/*v* at high frequencies

Using our model, we characterize the year in three stages and can now explain the mechanism of *dv*/*v* variations:

1. April to July: *dv*/*v* decreases sharply because the pore pressure increases rapidly when the snow meltwater enters the crust. The load on the underlying crust is unchanged as water moves from being snow above the surface to shallow groundwater. However, some water starts to drain from the system (sideways or deeper into the crust, below the region being sampled), and the consequent reduction in the elastic load also contributes to *dv*/*v* decreasing. The decrease in load from atmospheric pressure (starting around late June) also means that the rate of the decrease in load is slower than that of the decrease in snow thickness.

2. July to October: After all the snow has melted, there is no further meltwater input, so the GWL drops as water continues to drain away. This causes the pore pressure to decrease (increasing the velocity), but the load also continues to decrease (decreasing the velocity). This explains why *dv*/*v* increases, but at a slower rate than the decrease in GWL. Once all the water has drained away, the pore pressure returns to base levels, but the load, and therefore *dv*/*v*, is not yet back to its maximum.

3. October to April: As snow accumulates over the course of the winter, the load increases back to its maximum, while the GWL (and pore pressure) remains roughly constant, so *dv*/*v* increases back to its maximum value.

Last, we consider the seasonal variation of *dv*/*v* in the intermediate frequency bands: 0.4−1.0 and 1−2 Hz. As shown in Fig. 4 (A and B), *dv*/*v* in the 0.4–1 Hz band (~0- to 3-km depth) drops at the same time as in the higher frequency bands but generally stays low throughout the summer, only starting to increase again around November, rather than exhibiting the partial recovery in July and August seen at higher frequencies. The *dv*/*v* trend in the 1–2 Hz band (~0- to 1.5-km depth) generally falls between those at 0.4−1.0 and 2−4 Hz. The timing of the *dv*/*v* drop (while elastic load remains unchanged; Fig. 4) clearly demonstrates that the effect of elevated pore pressure is still important at these intermediate depths (~0 to 3 km). However, to explain the different shape of the yearly *dv*/*v* trend, we suggest that unlike in the shallowest crust (where pore pressure is most closely tied to GWL), pore pressure over this depth range remains elevated for some time after the yearly snowmelt around May. This may be because it takes significant time for water to percolate deeper into the crust than the region being sampled (i.e., behavior that is not captured in our simple exponential-outflow model for GWL) or because it is effectively stored here through the summer as it gradually flows laterally toward the sea. Because of the uncertainty in the permeability structure (see the Supplementary Materials) and groundwater flow paths across the region, we do not attempt to model this effect further.

An important caveat is that we have assumed that the depth sensitivity of the coda of the NCFs can be approximated using the sensitivity kernels of Rayleigh waves to simplify our analysis [e.g., (*37*)]. We however acknowledge that Love waves likely also contribute and that body waves may dominate at higher frequencies, particularly later in the coda (*38*). For the purposes of our study, the assumption that *dv*/*v* changes at higher frequencies represent changes in the shallower structure is the key aspect for our interpretation.

### Permeability structure of the Icelandic crust

The results of a magnetotelluric survey in our study area suggest that the shallowest ∼1 km of crust is highly permeable, with strong groundwater flow (*39*). This is underlain by a layer with much lower permeability, similar to the cap rocks covering high-temperature geothermal fields in Iceland, due to abundant clay mineralization. We therefore envisage that the pore pressure varies over short timescales in the shallow high-permeability layer, closely matching the GWL as measured in the shallow borehole. In fig. S12, we model pore pressure using a one-dimensional diffusion approach (*14*, *35*, *36*) and show that the time series of shallow pore pressure changes is very similar to that of measured GWL. This layered permeability structure could also explain why the effects of varying pore pressure on *dv*/*v* as indicated by GWL are negligible in the 0.1−0.4 Hz frequency band. The depth sensitivity in this case is ~1 to 8 km, i.e., over relatively impermeable rocks. Very little of the water input at the surface will reach these depths, and if it does, the evolution of pore pressure through time due to fluid percolation would be very different to the time series of GWL. The porosity is presumably also low, and poroelastic effects will therefore be greatly reduced. Instead, given the clear positive correlation between the total load and *dv*/*v* in the 0.1−0.4 Hz frequency band, elastic loading is considered to be the primary control.

### Estimating stress sensitivity from seasonal variations

We have shown that elastic loading can explain the seasonal variations in *dv*/*v* for the 0.1−0.4 Hz frequency band, whereas a combination of effects from loading and changing GWL is necessary to explain variation in *dv*/*v* in the 2−4 Hz band. Using the coefficients of load from Eqs. 4 and 5, we can estimate the stress sensitivity of *dv*/*v*. For 0.1−0.4 Hz, we find the coefficient of load to be 0.07%/m, corresponding to a velocity-stress sensitivity of ~7 × 10^−8^/Pa; for 2–4 Hz, the coefficient of 1.1%/m corresponds to 10^−6^/Pa. Both are within the range of values collated by Yamamura *et al*. (*30*). These stress sensitivities are several orders of magnitude larger than the stress sensitivity found from the dike response, affecting the same medium, in the 0.4–1.0 Hz band (~4 × 10^−9^/Pa); however, as discussed above, this is likely to be an underestimate.

As was observed with the changes in *dv*/*v* after the dike intrusion, the lower stress sensitivity at greater depths suggests that the crust at greater depths is less cracked with a lower porosity and so is less compliant. Note that the velocity profile in fig. S1 also illustrates this; the velocity gradient decreases with increasing depth as the crust becomes less porous and cracked, causing the effect of incrementally increasing confining pressure on velocity to decrease. Our observations are in agreement with the findings of Takano *et al*. (*40*), who seek to explain this phenomenon with numerical simulations of velocity-stress sensitivity using a granular model of the crust. We are fortunate in our study that we are able to investigate the relationship between velocity-stress sensitivity and depth using measurements of the response of the same volume of crust, over the same time period and using the same network, to two very different stress fields. This provides more robust evidence for this phenomenon than from the compilation of stress sensitivities from a range of seismic experiments (*40*). We explore this further with a forward model for *dv*/*v* changes under an increase in confining pressure equivalent to the seasonal elastic load (see the Supplementary Materials) and find encouraging agreement. This raises the exciting question of whether it may be possible to use this relationship in future to make first-order estimates of the stress change responsible for *dv*/*v* changes measured at a given frequency/depth.

### Other possible mechanisms to explain the seasonal variation

We have shown that changes in GWL result in changes in *dv*/*v* and have suggested that pore pressure variations are the cause of this relationship. However, varying fluid saturations may also play a role. Generally, P wave velocity increases with saturation and *S* wave velocity decreases (*41*). It is possible that the NCF coda (potentially composed of Rayleigh, Love, and body waves) is dominated by S waves, meaning that higher GWL (and greater fluid saturation) would result in a decrease in *dv*/*v* (the same relationship as with pore pressure). However, P waves likely also play a significant role, so this is clearly a complex relationship; we chose to use GWL in our model because it is a measurable metric that incorporates both effects.

Varying fluid saturations may also modulate the velocity-stress sensitivity of the crust through the year. Silver *et al*. (*19*) find that *dv*/*v* at two sites, one near a water well and one further away, respond oppositely to atmospheric pressure loading and attribute this to differences in fluid saturation between the two locations. Our model therefore could be developed by allowing the coefficients of elastic load and pore pressure (*A* and *B*, Eq. 5) to vary through the year; we chose not to do this because it would introduce further unknowns without sufficient constraint.

Frost has also been found to cause *dv*/*v* to increase during the winter (*18*); freezing water in pore spaces within a rock will greatly increase its rigidity (*42*), leading to increased seismic velocities. However, we observe *dv*/*v* starting to drop before the onset of thawing, as measured by the internal thermistors within the seismometers (fig. S13). Furthermore, the ground only freezes to ~0.5-m depth (approximately the same as the maximum burial depth of our instruments) each year at these altitudes (*43*). If freezing were an important mechanism, we would expect the amplitude of *dv*/*v* changes to be much greater at the highest frequencies, which are far more sensitive to these very shallow depths (fig. S1), but this is not observed (Fig. 4C). Another important consideration is that there is likely to be very little water persistently present in the shallowest 0.5 m of the extremely permeable cracked fresh lavas of the Northern Volcanic Zone, in contrast to the peaty soil above permafrost found in Alaska (*18*). This supposition is supported by very high resistivities measured at the shallowest depths in a magnetotelluric survey within the study region (*39*).

Changes in the persistent noise source, such as its spatial distribution and frequency content, could potentially contaminate the measurement of *dv*/*v* (*44*), particularly considering that the oceanic microseism also changes seasonally in the North Atlantic (*45*). However, *dv*/*v* reported here is likely to be a robust measurement of true velocity changes in the crust for several reasons. First, we measure changes in the coda of the NCFs, which sample the medium densely and have low sensitivity to noise source changes (*46*). We find that the same *dv*/*v* variations are also measured even later in the coda (fig. S5). Furthermore, we compare *dv*/*v* with a spectrogram of the seasonally varying secondary oceanic microseism (fig. S6) and can see that the changes are out of phase. Last, we measure a similar signal across a wide range of frequency bands and for many pairs of stations separated by different azimuths (fig. S4). That the seasonal *dv*/*v* signal in the 0.1−0.4 Hz band (encompassing the secondary oceanic microseism) is out of phase with the measurements at higher frequencies could be an indication of the influence of a changing noise source, although this too is out of phase with the changes visible in the spectrogram in fig. S6.

## CONCLUSIONS

We observe a change in *dv*/*v* after the Bárðarbunga-Holuhraun dike intrusion in 2014. By measuring *dv*/*v* between pairs of components at individual stations surrounding the dike, we show a linear relationship with volumetric strain across both positive and negative strain changes. The fact that there can be opposite changes in *dv*/*v* during the same event is an important consideration when using *dv*/*v* to study deformation associated with the often complex stress fields at volcanoes, particularly if measurements are averaged over a network of stations.

Seasonal variations in snow thickness, atmospheric pressure, and GWL in the Northern Volcanic Zone have a pronounced effect on *dv*/*v*. By careful analysis of 10 years of *dv*/*v* time series across a range of frequencies, we propose a simple model to explain the observed seasonal signal. In the shallowest ~1 km, two factors contribute to *dv*/*v* changes: cracks opening and closing due to elastic loading from snow, atmospheric pressure, and groundwater, and changes in pore pressure caused by snowmelt and rainwater percolating into (and later draining from) the crust. At greater depths, elastic loading alone is sufficient to explain the seasonal variation in *dv*/*v*. Thus, by studying *dv*/*v* across a range of frequency bands, we are able to comment on the structure of fluid flow paths within the crust: seasonal pore pressure changes are significantly greater in the shallow crust (<~1 km) compared with greater depths (~1 to 8 km).

Our model could be further refined if the effects of frost, the layered permeability structure of the crust, lateral groundwater flow, glacial meltwater, ocean tides, and thermoelastic strain (*47*) were incorporated. This could be achieved if denser measurements of weather and geodetic data were available in the study area and if uncertainties in the shallow seismic velocity structure and fluid flow paths could be reduced.

This study builds on previous work showing that *dv*/*v* of the upper crust is sensitive to a wide variety of stress changes, including those from magmatic intrusions, environmental loads and varying pore pressure. The sensitivity of *dv*/*v* to very small stress changes (we obtain a velocity-stress sensitivity of ∼10^−6^/Pa at 2–4 Hz) shows its potential as a technique for monitoring a wide range of phenomena affecting the crust. Cross-correlation of ambient seismic noise means that *dv*/*v* can be measured continuously, even with only one seismic station, and complements other geophysical measurements, including those of surface deformation and microseismicity, to understand the dynamics of the shallow crust. *dv*/*v* could therefore be a useful tool for monitoring volcanoes both in Iceland and elsewhere, providing that the seasonal variations described here are accounted for. Moreover, it may also, for example, be extended to monitoring anthropogenic- and climate change–related GWL changes over similarly large areas.

## MATERIALS AND METHODS

### Measuring *dv*/*v*

We used data from 51 broadband, three-component seismometers across the Northern Volcanic Zone: 35 stations from the University of Cambridge’s network, 18 stations from the national network of the IMO, 2 from University College Dublin, and 1 from the British Geological Survey. The average station elevation is 700-m above sea level. The data were stored in day-long miniSEED files at 50 or 100 Hz.

We used the program MSNoise to measure *dv*/*v* in five frequency bands: 0.1–0.4 Hz, 0.4–1.0 Hz, 1–2 Hz, 2–4 Hz, and 4–16 Hz. Before filtering, the data were preprocessed by resampling to 10 Hz for the four lower frequency bands and 50 Hz for the higher frequency band. Here, we calculated NCFs between the different components at the same station [“single-station cross-components,” east-north (EN), east-vertical (EZ), and north-vertical (NZ)]. We also calculated NCFs between all nine component pairs of pairs of stations in the lowest four frequency bands (results shown in fig. S4). Before cross-correlation, the waveforms were temporally normalized by clipping values higher than three times the root-mean-square amplitude and were spectrally whitened in 30-min windows.

For the frequency bands 0.1–0.4 and 0.4–1.0 Hz, we stacked NCFs over the stable time period up to 15 August 2014, the day before the Bárðarbunga-Holuhraun rifting event started. The intense seismicity during the rifting event alters the NCFs (Fig. 2), so we chose not to measure *dv*/*v* during this time, as the results were probably unreliable. For the frequency bands 1–2 Hz, 2–4 Hz, and 4–16 Hz, initial measurements showed that the magnitude of changes to the arrival times in the NCFs over a year was comparable to the dominant period of the NCFs. We therefore followed the methodology of James *et al*. (*18*) and used moving reference functions. *dv*/*v* was measured daily between two adjacent 5-day stacks (1 day apart), and *dv*/*v* was then summed cumulatively through time. Further details are given in the Supplementary Materials.

We used both the stretching (*26*, *48*) and MWCS (*49*) methods to calculate changes in the arrival times of phases in the coda of the NCFs relative to the reference functions. The MWCS method is less susceptible to spuriously measuring changes in the noise source (*44*), but the stretching method is more stable when the signal-to-noise ratio is high (*50*). We measured changes in phase in different time windows in the NCFs depending on the frequency band (see table S1 for all parameters), but always in the coda, defined as the section of the NCF that is linear when the log of the envelope of amplitude is plotted against time (fig. S5). We rejected any measurements of *dv/v* when the correlation coefficient between the stretched NCF and the reference NCF was below 0.4.

### Depth sensitivity kernels

We used Computer Programs in Seismology to calculate Rayleigh wave phase velocity depth sensitivity kernels. We used the velocity model from (*21*) for depths greater than 500 m below the surface. Since the sensitivity kernels of surface waves can be significantly affected by the shallowest structure (*51*) and earthquake tomography is relatively insensitive to these depths, we used a generic velocity model for the shallow velocity structure at volcanoes (*52*) for the top 500 m. The coarseness of our velocity model at very shallow depths limits the resolution of the kernels that we calculate in the highest frequency bands.

## Supplementary Material

http://advances.sciencemag.org/cgi/content/full/5/11/eaax6642/DC1

Download PDF

Crustal seismic velocity responds to a magmatic intrusion and seasonal loading in Iceland’s Northern Volcanic Zone

## SUPPLEMENTARY MATERIALS

Supplementary material for this article is available at http://advances.sciencemag.org/cgi/content/full/5/11/eaax6642/DC1 Section S1. Robustness of *dv/v* results Section S2. Choice of reference functions Section S3. Lateral sensitivity of NCF coda waves Section S4. Spatial variations in *dv/v* Section S5. Comparison of measured and modeled GWL Section S6. Forward model of changes in *dv/v* from Rayleigh wave phase velocities Section S7. Modeling pore pressure variations Section S8. Seasonal variation in *dv/v* and frost Fig. S1. Depth sensitivity kernels. Fig. S2. Analysis of changes in *dv/v* before and after the dike intrusion across different frequency bands. Fig. S3. Comparison of MWCS and stretching *dv/v* results. Fig. S4. Comparison of *dv/v* results from station pairs and single-station cross-components. Fig. S5. Comparison of *dv/v* results from different time lags in the NCFs. Fig. S6. Comparison of *dv/v* results with the frequency content and amplitude of the noise source. Fig. S7. Comparison of *dv/v* measurements using static references and moving references. Fig. S8. Frequency content with lag time of an NCF at FLUR in the frequency band 0.4−1.0 Hz. Fig. S9. Spatial variations in *dv/v* at 0.4−1.0 Hz. Fig. S10. Comparison of measured and modeled GWL. Fig. S11. Model of seasonal variations in *dv/v* at station SVA. Fig. S12. Comparison of pore pressure and GWL models. Fig. S13. Comparison of *dv/v* and temperature data. Table S1. MSNoise parameters References (*53*–*58*)

## References

[1] Brenguier F., Rivet D., Obermann A., Nakata N., Boué P., Lecocq T., Campillo M., Shapiro N., 4-D noise-based seismology at volcanoes: Ongoing efforts and perspectives. J. Volcanol. Geotherm. Res. 321, 182–195 (2016).

[2] Sens-Schönfelder C., Pomponi E., Peltier A., Dynamics of Piton de la Fournaise volcano observed by passive image interferometry with multiple references. J. Volcanol. Geotherm. Res. 276, 32–45 (2014).

[3] Donaldson C., Caudron C., Green R. G., Thelen W. A., White R. S., Relative seismic velocity variations correlate with deformation at Kīlauea volcano. Sci. Adv. 3, e1700219 (2017). 28782009 10.1126/sciadv.1700219 PMC5489268

[4] Ueno T., Saito T., Shiomi K., Enescu B., Hirose H., Obara K., Fractional seismic velocity change related to magma intrusions during earthquake swarms in the eastern Izu peninsula, central Japan. J. Geophys. Res. Solid Earth. 117, (2012).

[5] Caudron C., Lecocq T., Syahbana D. K., McCausland W., Watlet A., Camelbeeck T., Bernard A., Surono, Stress and mass changes at a “wet” volcano: Example during the 2011–2012 volcanic unrest at Kawah Ijen volcano (Indonesia). J. Geophys. Res. Solid Earth. 120, 2014JB011590 (2015).

[6] Brenguier F., Shapiro N. M., Campillo M., Ferrazzini V., Duputel Z., Coutant O., Nercessian A., Towards forecasting volcanic eruptions using seismic noise. Nat. Geosci. 1, 126–130 (2008).

[7] Sigmundsson F., Hooper A., Hreinsdóttir S., Vogfjörd K. S., Ófeigsson B. G., Heimisson E. R., Dumont S., Parks M., Spaans K., Gudmundsson G. B., Drouin V., Árnadóttir T., Jónsdóttir K., Gudmundsson M. T., Högnadóttir T., Fridriksdóttir H. M., Hensch M., Einarsson P., Magnússon E., Samsonov S., Brandsdóttir B., White R. S., Ágústsdóttir T., Greenfield T., Green R. G., Hjartardóttir Á. R., Pedersen R., Bennett R. A., Geirsson H., La Femina P. C., Björnsson H., Pálsson F., Sturkell E., Bean C. J., Möllhoff M., Braiden A. K., Eibl E. P. S., Segmented lateral dyke growth in a rifting event at Bárðarbunga volcanic system, Iceland. Nature 517, 191–195 (2015). 25517098 10.1038/nature14111

[8] Woods J., Winder T., White R. S., Brandsdóttir B., Evolution of a lateral dike intrusion revealed by relatively-relocated dike-induced earthquakes: The 2014–15 Bárðarbunga–Holuhraun rifting event, Iceland. Earth Planet. Sci. Lett. 506, 53–63 (2019).

[9] Ruch J., Wang T., Xu W., Hensch M., Jónsson S., Oblique rift opening revealed by reoccurring magma injection in central Iceland. Nat. Commun. 7, 12352 (2016). 27492709 10.1038/ncomms12352 PMC4980445

[10] Pedersen G. B. M., Höskuldsson A., Dürig T., Thordarson T., Jónsdóttir I., Riishuus M. S., Óskarsson B. V., Dumont S., Magnússon E., Gudmundsson M. T., Sigmundsson F., Drouin V. J. P. B., Gallagher C., Askew R., Gudnason J., Moreland W. M., Nikkola P., Reynolds H. I., Schmith J., Lava field evolution and emplacement dynamics of the 2014-2015 basaltic fissure eruption at Holuhraun, Iceland. J. Volcanol. Geotherm. Res. 340, 155–169 (2017).

[11] Woods J., Donaldson C., White R. S., Caudron C., Brandsdóttir B., Hudson T. S., Ágústsdóttir T., Long-period seismicity reveals magma pathways above a laterally propagating dyke during the 2014–15 Bárðarbunga rifting event, Iceland. Earth Planet. Sci. Lett. 490, 216–229 (2018).

[12] Gudmundsson M. T., Jónsdóttir K., Hooper A., Holohan E. P., Halldórsson S. A., Ófeigsson B. G., Cesca S., Vogfjörd K. S., Sigmundsson F., Högnadóttir T., Einarsson P., Sigmarsson O., Jarosch A. H., Jónasson K., Magnússon E., Hreinsdóttir S., Bagnardi M., Parks M. M., Hjörleifsdóttir V., Pálsson F., Walter T. R., Schöpfer M. P. J., Heimann S., Reynolds H. I., Dumont S., Bali E., Gudfinnsson G. H., Dahm T., Roberts M. J., Hensch M., Belart J. M. C., Spaans K., Jakobsson S., Gudmundsson G. B., Fridriksdóttir H. M., Drouin V., Dürig T., Adalgeirsdóttir G., Riishuus M. S., Pedersen G. B. M., van Boeckel T., Oddsson B., Pfeffer M. A., Barsotti S., Bergsson B., Donovan A., Burton M. R., Aiuppa A., Gradual caldera collapse at Bárdarbunga volcano, Iceland, regulated by lateral magma outflow. Science 353, (2016). 10.1126/science.aaf8988 27418515

[13] Clements T., Denolle M. A., Tracking Groundwater Levels Using the Ambient Seismic Field. Geophys. Res. Lett. 45, 6459–6465 (2018).

[14] Wang Q.-Y., Brenguier F., Campillo M., Lecointre A., Takeda T., Aoki Y., Seasonal Crustal Seismic Velocity Changes Throughout Japan. J. Geophys. Res. Solid Earth. 122, 7987–8002 (2017).

[15] Richter T., Sens-Schönfelder C., Kind R., Asch G., Comprehensive observation and modeling of earthquake and temperature-related seismic velocity changes in northern Chile with passive image interferometry. J. Geophys. Res. Solid Earth. 119, 4747–4765 (2014).

[16] Hotovec-Ellis A. J., Gomberg J., Vidale J. E., Creager K. C., A continuous record of intereruption velocity change at Mount St. Helens from coda wave interferometry. J. Geophys. Res. Solid Earth. 119, 2013JB010742 (2014).

[17] Cannata A., Cannavò F., Montalto P., Ercoli M., Mancinelli P., Pauselli C., Leto G., Monitoring crustal changes at volcanoes by seismic noise interferometry: Mt. Etna case of study. J. Volcanol. Geotherm. Res. 337, 165–174 (2017).

[18] James S. R., Knox H. A., Abbott R. E., Screaton E. J., Improved moving window cross-spectral analysis for resolving large temporal seismic velocity changes in permafrost. Geophys. Res. Lett. 44, 4018–4026 (2017).

[19] Silver P. G., Daley T. M., Niu F., Majer E. L., Active source monitoring of cross-well seismic travel time for stress-induced changes. Bull. Seismol. Soc. Am. 97, 281–293 (2007).

[20] Drouin V., Heki K., Sigmundsson F., Hreinsdóttir S., Ófeigsson B. G., Constraints on seasonal load variations and regional rigidity from continuous GPS measurements in Iceland, 1997–2014. Geophys. J. Int. 205, 1843–1858 (2016).

[21] Greenfield T., White R. S., Winder T., Ágústsdóttir T., Seismicity of the Askja and Bárðarbunga volcanic systems of Iceland, 2009–2015. J. Volcanol. Geotherm. Res., in press (2018).

[22] Plaen R. S. M. D., Lecocq T., Caudron C., Ferrazzini V., Francis O., Single-station monitoring of volcanoes using seismic ambient noise. Geophys. Res. Lett. 43, 8511–8518 (2016).

[23] Green R. G., Greenfield T., White R. S., Triggered earthquakes suppressed by an evolving stress shadow from a propagating dyke. Nat. Geosci. 8, 629–632 (2015).

[24] Drouin V., Sigmundsson F., Ófeigsson B. G., Hreinsdóttir S., Sturkell E., Einarsson P., Deformation in the Northern Volcanic Zone of Iceland 2008–2014: An interplay of tectonic, magmatic, and glacial isostatic deformation. J. Geophys. Res. Solid Earth. 122, 3158–3178 (2017).

[25] Nawri N., Pálmason B., Petersen G. N., Björnsson H., Þorsteinsson S., The ICRA atmospheric reanalysis project for Iceland. Icel Meteorol Off. 005 (2017).

[26] Sens-Schönfelder C., Wegler U., Passive image interferometry and seasonal variations of seismic velocities at Merapi Volcano, Indonesia. Geophys. Res. Lett. 33, (2006).

[27] Carrier A., Got J.-L., Peltier A., Ferrazzini V., Staudacher T., Kowalski P., Boissier P., A damage model for volcanic edifices: Implications for edifice strength, magma pressure, and eruptive processes. J. Geophys. Res. Solid Earth. 120, 567–583 (2015).

[28] Olivier G., Brenguier F., Carey R., Okubo P., Donaldson C., Decrease in Seismic Velocity Observed Prior to the 2018 Eruption of Kīlauea Volcano With Ambient Seismic Noise Interferometry. Geophys. Res. Lett. 46, 3734–3744 (2019).

[29] Parks M. M., Heimisson E. R., Sigmundsson F., Hooper A., Vogfjörd K. S., Árnadóttir T., Ófeigsson B., Hreinsdóttir S., Hjartardóttir Á. R., Einarsson P., Gudmundsson M. T., Högnadóttir T., Jónsdóttir K., Hensch M., Bagnardi M., Dumont S., Drouin V., Spaans K., Ólafsdóttir R., Evolution of deformation and stress changes during the caldera collapse and dyking at Bárdarbunga, 2014–2015: Implication for triggering of seismicity at nearby Tungnafellsjökull volcano. Earth Planet. Sci. Lett. 462, 212–223 (2017).

[30] Yamamura K., Sano O., Utada H., Takei Y., Nakao S., Fukao Y., Long-term observation of in situ seismic velocity and attenuation. J. Geophys. Res. Solid Earth. 108, (2003).

[31] Jonsson S., Segall P., Pedersen R., Björnsson G., Post-earthquake ground movements correlated to pore-pressure transients. Nature 424, 179–183 (2003). 12853953 10.1038/nature01776

[32] Snaebjörnsdóttir S. Ó., Wiese F., Fridriksson T., Ármansson H., Einarsson G. M., Gislason S. R., CO2 storage potential of basaltic rocks in Iceland and the oceanic ridges. Energy Procedia 63, 4585–4600 (2014).

[33] Einarsson P., Brandsdóttir B., Earthquakes in the Myrdalsjokull area, Iceland, 1978–1985; seasonal correlation and connection with volcanoes. Jokull. 49, 59–73 (2000).

[34] Obermann A., Froment B., Campillo M., Larose E., Planès T., Valette B., Chen J. H., Liu Q. Y., Seismic noise correlations to image structural and mechanical changes associated with the Mw 7.9 2008 Wenchuan earthquake. J. Geophys. Res. Solid Earth. 119, 3155–3168 (2014).

[35] Rivet D., Brenguier F., Cappa F., Improved detection of preeruptive seismic velocity drops at the Piton de La Fournaise volcano. Geophys. Res. Lett. 42, 6332–6339 (2015).

[36] Talwani P., Chen L., Gahalaut K., Seismogenic permeability, ks. J. Geophys. Res. Solid Earth. 112, 10.1029/2006JB004665, (2007).

[37] Taira T., Brenguier F., Response of hydrothermal system to stress transients at Lassen Volcanic Center, California, inferred from seismic interferometry with ambient noise. Earth Planets Space 68, 162 (2016).

[38] Obermann A., Planès T., Larose E., Sens-Schönfelder C., Campillo M., Depth sensitivity of seismic coda waves to velocity perturbations in an elastic heterogeneous medium. Geophys. J. Int. 194, 372–382 (2013).

[39] A. M. Vilhjálmsson, Ó. G. Flóvenz, “Geothermal Implications from a Resistivity Survey in the Volcanic Rift Zone of NE-Iceland and Comparison with Seismic Data” (Iceland Geosurvey, 2017).

[40] Takano T., Nishimura T., Nakahara H., Seismic velocity changes concentrated at the shallow structure as inferred from correlation analyses of ambient noise during volcano deformation at Izu-Oshima, Japan. J. Geophys. Res. Solid Earth 122, 6721–6736 (2017).

[41] Japsen P., Bruun A., Fabricius I. L., Rasmussen R., Vejbæk O. V., Pedersen J. M., Mavko G., Mogensen C., Høier C., Influence of porosity and pore fluid on acoustic properties of chalk: AVO response from oil, South Arne Field, North Sea. Pet. Geosci. 10, 319–330 (2004).

[42] Kneisel C., Hauck C., Fortier R., Moorman B., Advances in geophysical methods for permafrost investigations. Permafr. Periglac. Process. 19, 157–178 (2008).

[43] G. N. Petersen, D. Berber, “Jarðvegshitamælingar á Íslandi Staða núverandi kerfis og framtíðarsýn” (Veðurstofa Íslands, 2018).

[44] Zhan Z., Tsai V. C., Clayton R. W., Spurious velocity changes caused by temporal variations in ambient noise frequency content. Geophys. J. Int. ggt170 (2013).

[45] Sergeant A., Stutzmann E., Maggi A., Schimmel M., Ardhuin F., Obrebski M., Frequency-dependent noise sources in the North Atlantic Ocean. Geochem. Geophys. Geosystems. 14, 5341–5353 (2013).

[46] Colombi A., Chaput J., Brenguier F., Hillers G., Roux P., Campillo M., On the temporal stability of the coda of ambient noise correlations. Comptes Rendus Geosci. 346, 307–316 (2014).

[47] Meier U., Shapiro N. M., Brenguier F., Detecting seasonal variations in seismic velocities within Los Angeles basin from correlations of ambient seismic noise. Geophys. J. Int. 181, 985–996 (2010).

[48] Lobkis O. I., Weaver R. L., On the emergence of the Green’s function in the correlations of a diffuse field. J. Acoust. Soc. Am. 110, 3011–3017 (2001). 10.1121/1.1898683 16018447

[49] Poupinet G., Ellsworth W. L., Frechet J., Monitoring velocity variations in the crust using earthquake doublets: An application to the Calaveras Fault, California. J. Geophys. Res. Solid Earth. 89, 5719–5731 (1984).

[50] Hadziioannou C., Larose E., Coutant O., Roux P., Campillo M., Stability of monitoring weak changes in multiply scattering media with ambient noise correlation: Laboratory experiments. J. Acoust. Soc. Am. 125, 3688–3695 (2009). 19507951 10.1121/1.3125345

[51] Yang C., Li G., Niu F., Ben-Zion Y., Significant effects of shallow seismic and stress properties on phase velocities of Rayleigh Waves Up to 20 s. Pure Appl. Geophys. 176, 1255–1267 (2019).

[52] Lesage P., Heap M. J., Kushnir A., A generic model for the shallow velocity structure of volcanoes. J. Volcanol. Geotherm. Res. 356, 114–126 (2018).

[53] Brenguier F., Campillo M., Takeda T., Aoki Y., Shapiro N. M., Briand X., Emoto K., Miyake H., Mapping pressurized volcanic fluids from induced crustal seismic velocity drops. Science 345, 80–82 (2014). 24994652 10.1126/science.1254073

[54] Bennington N., Haney M., Thurber C., Zeng X., Inferring Magma Dynamics at Veniaminof Volcano Via Application of Ambient Noise. Geophys. Res. Lett. 45, 11,650–11,658 (2018).

[55] Obermann A., Lupi M., Mordret A., Jakobsdóttir S. S., Miller S. A., 3D-ambient noise Rayleigh wave tomography of Snæfellsjökull volcano, Iceland. J. Volcanol. Geotherm. Res. 317, 42–52 (2016).

[56] Tsai V. C., A model for seasonal changes in GPS positions and seismic wave speeds due to thermoelastic and hydrologic variations. J. Geophys. Res. Solid Earth. 116, 10.1029/2010JB008156, (2011).

[57] Rivet D., Campillo M., Shapiro N. M., Cruz-Atienza V., Radiguet M., Cotte N., Kostoglodov V., Seismic evidence of nonlinear crustal deformation during a large slow slip event in Mexico. Geophys. Res. Lett. 38, (2011).

[58] Chen L., Talwani P., Mechanism of initial seismicity following impoundment of the Monticello Reservoir, South Carolina. Bull. Seismol. Soc. Am. 91, 1582–1594 (2001).

