# Supplementary material for: Crustal seismic velocity responds to a magmatic intrusion and seasonal loading in Iceland’s Northern Volcanic Zone
Source: Sci Adv. 2019 Nov 27;5(11):eaax6642. doi: 10.1126/sciadv.aax6642 (PMC6881157; doi:10.1126/sciadv.aax6642)
Supplement: http://advances.sciencemag.org/cgi/content/full/5/11/eaax6642/DC1 [file supp_5_11_eaax6642__index.html]

Science Advances | Science AdvancesAAASSearchScience AdvancesMenu

## Supplementary Materials

**This PDF file includes:**

- Section S1. Robustness of *dv/v* results
- Section S2. Choice of reference functions
- Section S3. Lateral sensitivity of NCF coda waves
- Section S4. Spatial variations in *dv/v*
- Section S5. Comparison of measured and modeled GWL
- Section S6. Forward model of changes in *dv/v* from Rayleigh wave phase velocities
- Section S7. Modeling pore pressure variations
- Section S8. Seasonal variation in *dv/v* and frost
- Fig. S1. Depth sensitivity kernels.
- Fig. S2. Analysis of changes in *dv/v* before and after the dike intrusion across different frequency bands.
- Fig. S3. Comparison of MWCS and stretching *dv/v* results.
- Fig. S4. Comparison of *dv/v* results from station pairs and single-station cross-components.
- Fig. S5. Comparison of *dv/v* results from different time lags in the NCFs.
- Fig. S6. Comparison of *dv/v* results with the frequency content and amplitude of the noise source.
- Fig. S7. Comparison of *dv/v* measurements using static references and moving references.
- Fig. S8. Frequency content with lag time of an NCF at FLUR in the frequency band 0.4–1.0 Hz.
- Fig. S9. Spatial variations in *dv/v* at 0.4–1.0 Hz.
- Fig. S10. Comparison of measured and modeled GWL.
- Fig. S11. Model of seasonal variations in *dv/v* at station SVA.
- Fig. S12. Comparison of pore pressure and GWL models.
- Fig. S13. Comparison of *dv/v* and temperature data.
- Table S1. MSNoise parameters
- References (*53*–*58*)

Download PDF

**Files in this Data Supplement:**

- Adobe PDF - aax6642\_SM.pdf
