# Supplementary material for: Crustal seismic velocity responds to a magmatic intrusion and seasonal loading in Iceland’s Northern Volcanic Zone
Source: Sci Adv. 2019 Nov 27;5(11):eaax6642. doi: 10.1126/sciadv.aax6642 (PMC6881157; doi:10.1126/sciadv.aax6642)
Supplement: Download PDF [file aax6642_SM.pdf]

Science Advances
AAAS

advances.sciencemag.org/cgi/content/full/5/11/eaax6642/DC1

Supplementary Materials for

Crustal seismic velocity responds to a magmatic intrusion and seasonal loading in Iceland's Northern Volcanic Zone

C. Donaldson*, T. Winder*, C. Caudron, R. S. White

*Corresponding author. Email: cgd27@esc.cam.ac.uk (C.D.); tom.winder@esc.cam.ac.uk (T.W.)

Published 27 November 2019, *Sci. Adv.* **5**, eaax6642 (2019)
DOI: 10.1126/sciadv.aax6642

This PDF file includes:

Section S1. Robustness of *dv/v* results
Section S2. Choice of reference functions
Section S3. Lateral sensitivity of NCF coda waves
Section S4. Spatial variations in *dv/v*
Section S5. Comparison of measured and modeled GWL
Section S6. Forward model of changes in *dv/v* from Rayleigh wave phase velocities
Section S7. Modeling pore pressure variations
Section S8. Seasonal variation in *dv/v* and frost
Fig. S1. Depth sensitivity kernels.
Fig. S2. Analysis of changes in *dv/v* before and after the dike intrusion across different frequency bands.
Fig. S3. Comparison of MWCS and stretching *dv/v* results.
Fig. S4. Comparison of *dv/v* results from station pairs and single-station cross-components.
Fig. S5. Comparison of *dv/v* results from different time lags in the NCFs.
Fig. S6. Comparison of *dv/v* results with the frequency content and amplitude of the noise source.
Fig. S7. Comparison of *dv/v* measurements using static references and moving references.
Fig. S8. Frequency content with lag time of an NCF at FLUR in the frequency band 0.4–1.0 Hz.
Fig. S9. Spatial variations in *dv/v* at 0.4–1.0 Hz.
Fig. S10. Comparison of measured and modeled GWL.
Fig. S11. Model of seasonal variations in *dv/v* at station SVA.
Fig. S12. Comparison of pore pressure and GWL models.
Fig. S13. Comparison of *dv/v* and temperature data.
Table S1. MSNoise parameters.
References (*53–58*)

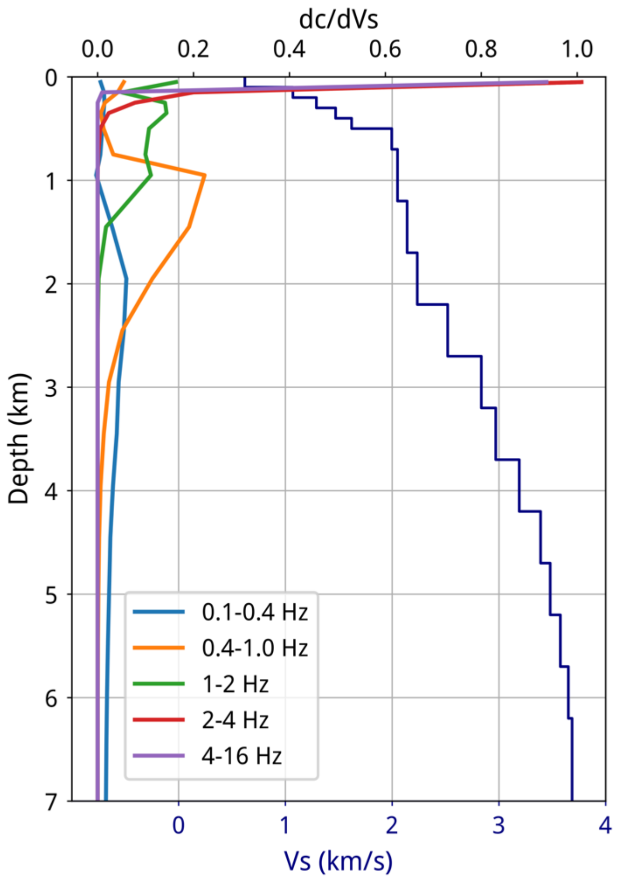

Fig. S1. Depth sensitivity kernels. Using the shear-wave velocity model shown in dark blue, phase-velocity sensitivity kernels of Rayleigh waves are estimated (see Materials and Methods). The kernels are calculated over a range of frequencies, then averaged within each of the five frequency bands used in this study.

<u>dv/v changes after the Bárðarbunga-Holuhraun rifting event for all frequency bands</u>

As discussed in the main text, it is inappropriate to use a static reference function (calculated by stacking data over a period of several years) for the higher frequency bands, because the amplitude of the seasonal changes in dv/v (and therefore dt/t) are large compared to the period of the NCFs. We therefore cannot use the same method to measure any step-changes in dv/v associated with the dike intrusion as for the 0.4-1 Hz frequency band. To make continuous measurements of dv/v at high frequencies (to study the seasonal variation in dv/v) we use a moving reference function technique (described further below). However, it is not possible to measure dv/v changes across a gap in measurements with this method, as is the case over the course of the Bárðarbunga-Holuhraun rifting event. Instead, we make direct measurements of dv/v between stacks of NCFs before and after the rifting event. For example, we stack all available data for January before the rifting event (to make one NCF) and after (to make a second NCF) for each cross-component pair at each station. If the correlation coefficient between the two NCFs is higher than 0.4, we measure dv/v between them (using the stretching technique). We do this for each month except May (when there is generally a large drop in dv/v, meaning the stacking may be incoherent) and then calculate the average and standard deviation of the 11 measurements. The results are plotted in fig. S2, using this method for all four frequency bands shown.

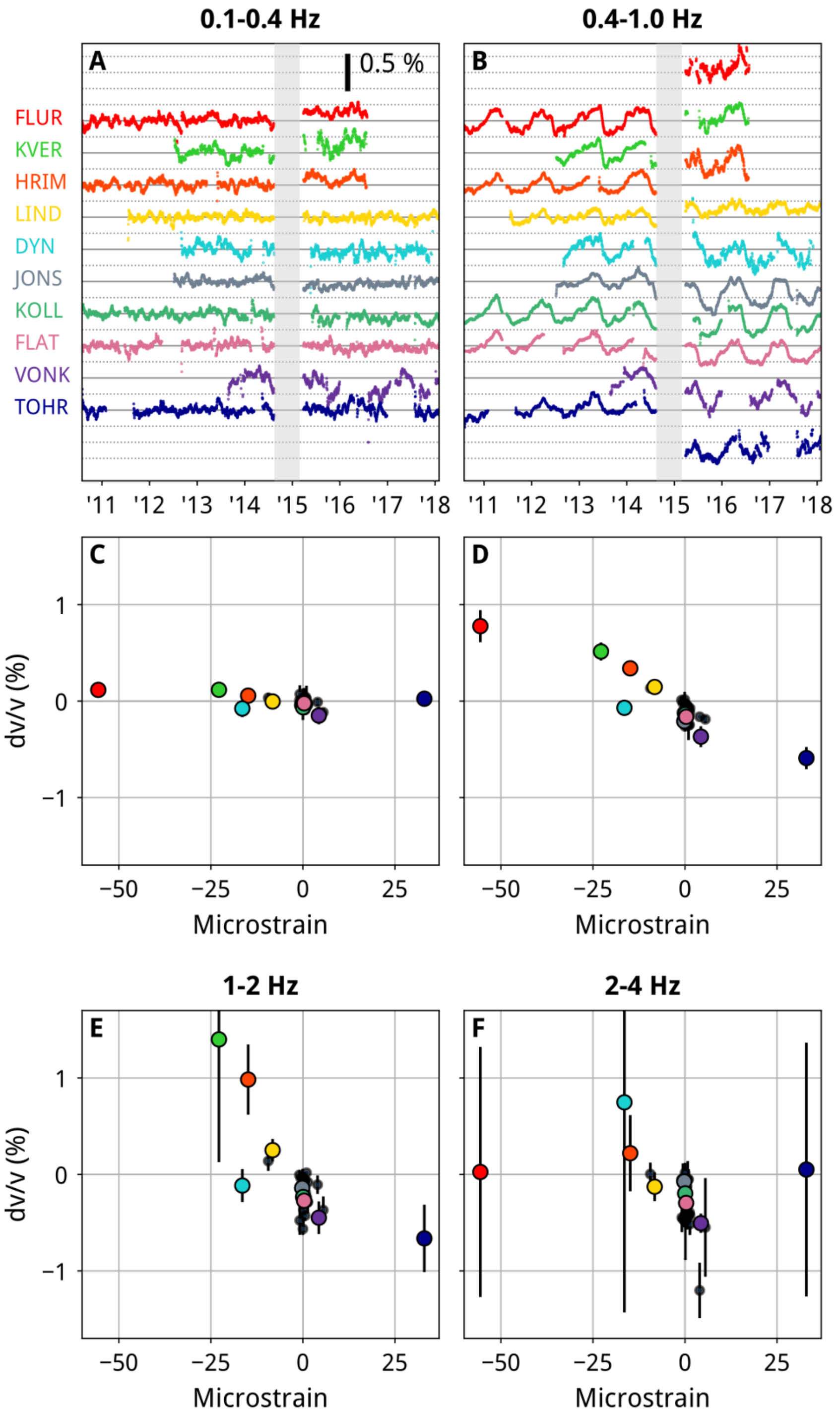

Fig. S2. Analysis of changes in *dv/v* before and after the dike intrusion across different frequency bands. (**A**) dv/v measurements from a selection of individual stations in 0.1−0.4 Hz band (average of EZ, NZ, EN components, 30-day stacks). (**B**) As in Fig. 3, dv/v measurements from a selection of individual stations in 0.4−1.0 Hz band (average of EZ, NZ, EN components, 30-day stacks). (**C**−**F**) For the frequency bands: (C) 0.1−0.4 Hz, (D) 0.4−1.0 Hz, (E) 1−2 Hz and (F) 2−4 Hz, measurements of dv/v are made for each month (excluding May, when there is a steep drop in dv/v) by stacking all available data for that month before and after the Bárðarbunga-Holuhraun rifting event for each component-pair (EZ, NZ, EN). If the correlation coefficient between the NCF before and the NCF after is less than 0.4 the result is rejected. The average and standard deviation of the dv/v measurements is calculated at each station and plotted against volumetric strain, as in Fig. 3.

Section S1. Robustness of *dv/v* results

In figs. S3−S6 we show the robustness of the measurements of dv/v. Results in this paper are consistent with those measured from noise cross-correlation functions (NCFs) calculated using the moving-window cross-spectral (MWCS) method (fig. S3) and between pairs of stations (fig. S4). Figure S5 shows that dv/v measured in later time windows in the NCFs are consistent.

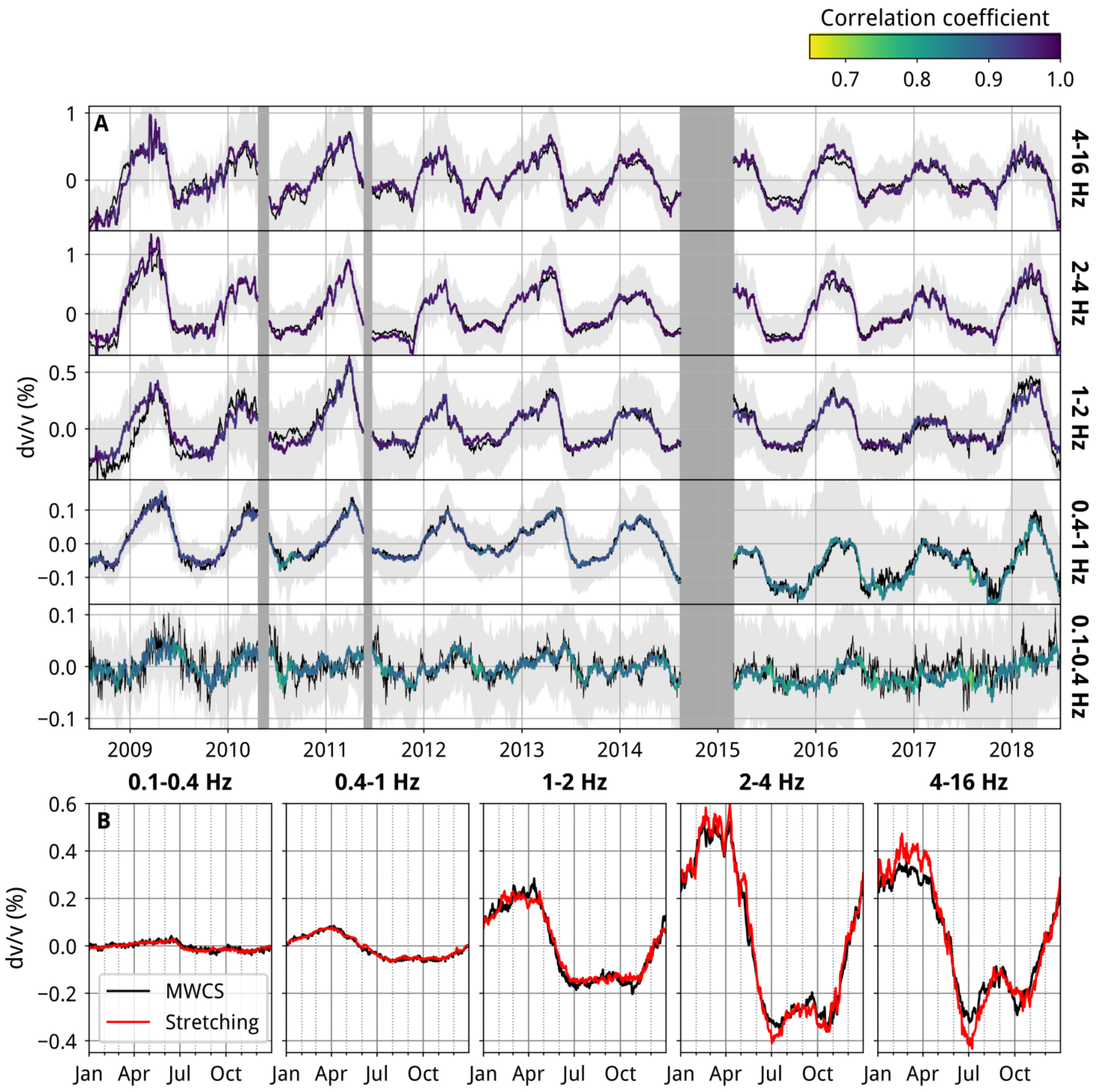

Fig. S3. Comparison of MWCS and stretching *dv/v* results. (**A**) dv/v measured using stretching (colored) and MWCS (gray) for the different frequency bands. The standard deviation in dv/v (stretching) measured at all stations across the network is shaded gray. dv/v is measured by comparing 30-day stacks to a single reference function for the lowest two frequency bands, and between pairs of 5-day stacks offset by one day – with the earlier stack acting as a moving reference function – for the three higher frequency bands. (**B**) Yearly averages of the same results. Note that the y-axis scales vary in (A) but are the same in (B).

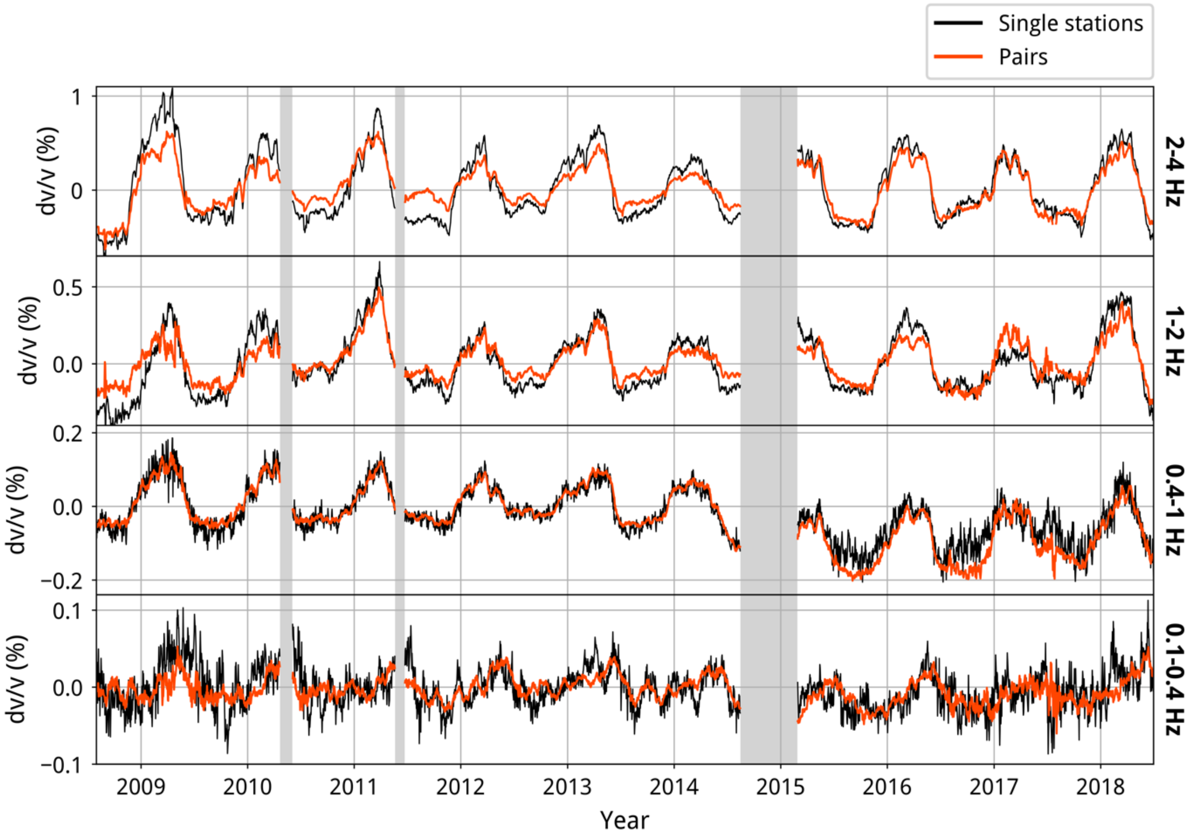

Fig. S4. Comparison of *dv/v* results from station pairs and single-station cross-components. dv/v measured using the MWCS method. All 9 component-pairs for pairs with an inter-station distance of less than 40 km (lower two frequency bands) or 15 km (higher two frequency bands) are averaged. Stacking and reference functions as in fig. S3. The windows used in the NCFs depend on the inter-station distance; the start of the window is the inter-station distance divided by 1 km/s, the width is the same as for the main results (see table S1).

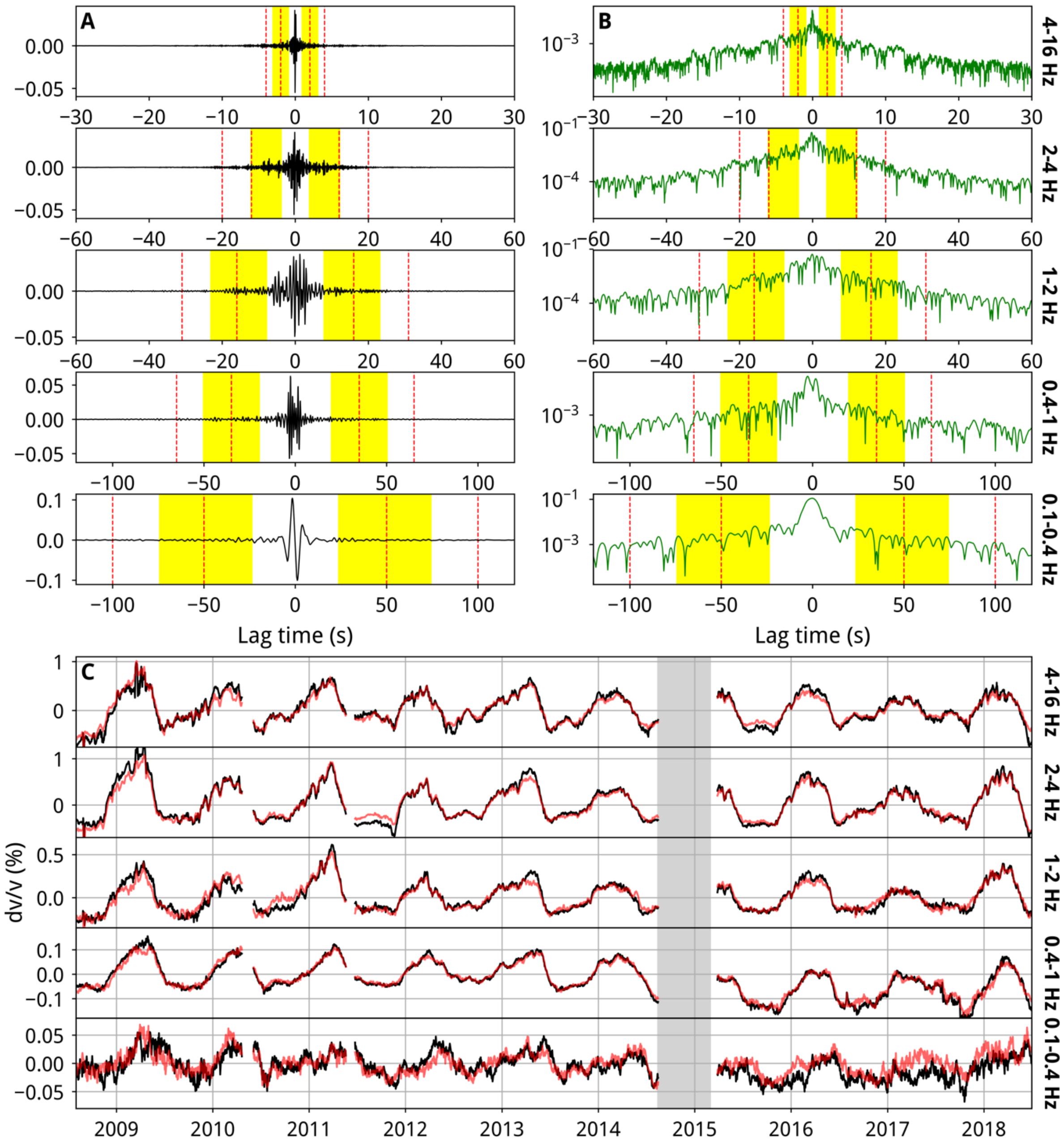

Fig. S5. Comparison of *dv/v* results from different time lags in the NCFs. (**A**) Stacks of NCFs until 15 August 2014 for station LIND, N-Z component pair, for all frequency bands. (**B**) Envelope of the NCFs in (A), plotted with a logarithmic y-scale. The coda is defined as the approximately-linear section of the envelope, after the ballistic arrival but before it flattens out. (**C**) Network-averaged dv/v (as in Fig. 4): black line corresponds to measurements made in the time lags highlighted by the yellow bars in (A) and (B); red line to the time lags shown by dashed red lines in (A) and (B). Further details of the time lags used in this study are given in table S1.

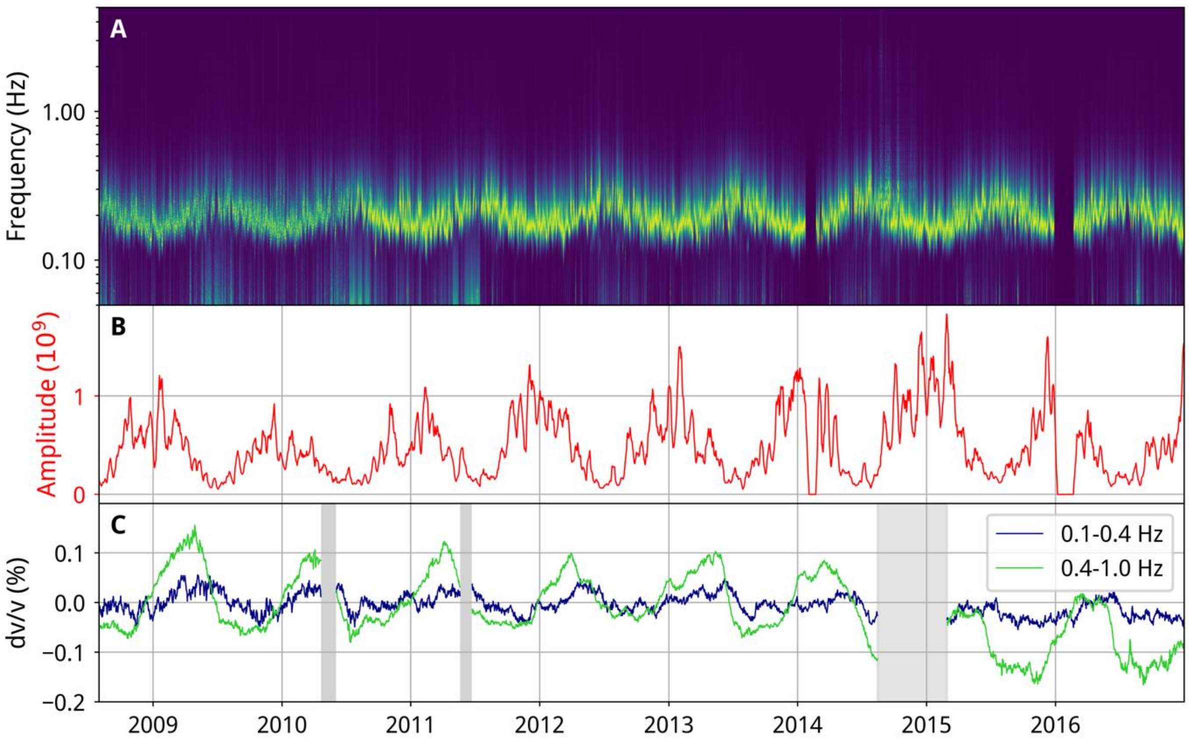

Fig. S6. Comparison of *dv/v* results with the frequency content and amplitude of the noise source. (**A**) Spectrogram of continuous seismic data recorded at station KOLL. Data is resampled to 20 Hz, split into 10-minute windows, the fast-fourier transform calculated in each window and an average spectrum determined each day, then normalized each day. (**B**) Amplitude between 0.1–1.0 Hz, as summed from the spectrogram in (A). (**C**) Network-averaged dv/v results in 0.1–0.4 Hz and 0.4–1.0 Hz frequency bands.

Section S2. Choice of reference functions

Ideally a reference noise cross-correlation function (NCF) is a stack over a representative, stable period of time. For the frequency bands 0.1−0.4 Hz and 0.4−1.0 Hz we used all available NCFs before 16 August 2014, when the Bárðarbunga-Holuhraun dyke intrusion began. Stacking over a long period of time means that the signal-to-noise ratio of the reference NCF is greater. However, this approach is less appropriate at higher frequencies. In this case, the changes in arrival time of the phases in the NCFs through time are comparable to the period of the NCFs themselves, so the NCFs may not stack constructively. Following the method of James *et al.* (*18*), we instead used moving reference functions for the frequency bands 1−2 Hz, 2−4 Hz and 4−16 Hz. We stacked 5-day periods and made daily measurements of dv/v between adjacent 5-day stacks offset by one day, with the first acting as a moving reference function. dv/v was then summed cumulatively through time.

This method has the advantage that the correlation coefficient between the current and reference NCF is consistently higher (fig. S7), so it is possible to achieve better time resolution. The disadvantage, as described by James *et al.*, is that any error in the measurement of dv/v propagates through the time series, since dv/v is a cumulative sum. It is not necessarily possible to identify times when the error is introduced, which could be at a single occurrence or a consistent bias over a period

of time. The effects of this error propagation can be seen as a long-term drift occurring in dv/v. Like James *et al.*, we correct for this drift by removing linear trends (one before the Bárðarbunga-Holuhraun rifting event and one afterwards) from the cumulatively summed dv/v time series for each component-pair at each individual station. This correction is empirical and means that long-term trends in dv/v cannot be interpreted from these results.

In fig. S7, we show that, in the 1−2 Hz band, the moving-reference results are consistent with results calculated using a single reference function stacked before the Bárðarbunga-Holuhraun rifting event (as was done for the lower frequency bands). For the two highest frequency bands, the timing of the annual cycle in dv/v is the same, but the amplitude of the trend is much greater when using the moving-reference method. This shows the importance of the choice of reference function, particularly at higher frequencies. An alternative to the moving-reference approach is the method of Brenguier *et al.* (*53*), where dv/v is measured between all combinations of days and a Bayesian least-squares inversion is used to determine a continuous dv/v time series.

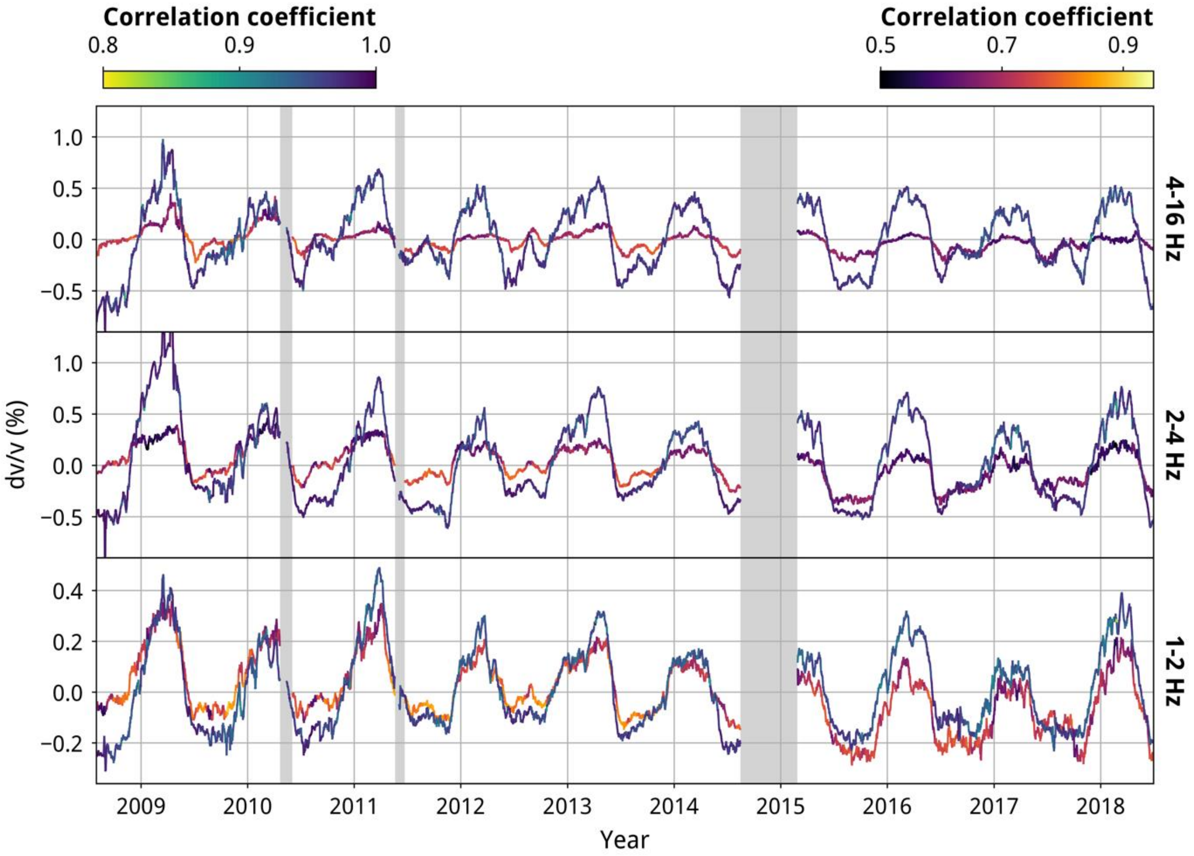

Fig. S7. Comparison of *dv/v* measurements using static references and moving references. Static references are stacks of all available data until 15 August 2014 (dv/v shown in black to yellow color palette); the current NCFs are 10-day stacks for 1–2 Hz and 2–4 Hz and 30-day stacks for 4–16 Hz. The moving references are the preceding 5-day stack (dv/v shown in yellow to purple color palette), see text for details. As in Fig. 4, dv/v is measured between the three component-pairs at individual stations and averaged over the network.

Section S3. Lateral sensitivity of NCF coda waves

To estimate the lateral sensitivity of the coda of the NCFs, we followed the approach of Bennington *et al.* (*54*); the reader is referred to their Supplementary Materials for a detailed explanation of the approach. We focus on the frequency band 0.4−1.0 Hz, because we are primarily interested in the smearing of the sensitivity at each station when studying the changes in dv/v before and after the 2014 dike intrusion. To approximate the lateral sensitivity, we can use the radius, *R*, of the first Fresnel zone for a source-receiver pair with zero-offset

$$R = \frac{v}{2}\sqrt{t_0 T}$$

where v is velocity, t_0 is two-way travel time and T is period. Because we are considering the coda of the NCF, the two-way travel time refers to the travel time from the station to an average scatterer and back again. By applying a series of narrow-band Gaussian filters to the NCF (a frequency-time analysis, FTAN) over the frequency band of interest (0.4−1.0 Hz), it is possible to pick the group travel time at the maximum amplitude. This is similar to picking dispersion curves in surface wave tomography; however, in this case, we are interested in the peak in the coda (not the direct surface wave arrival). Figure S8 shows the FTAN of an NCF at station FLUR. A peak in amplitude is visible at approximately -12 to -19 s across the frequency band, which may correspond to the arrival from the average scatterer.

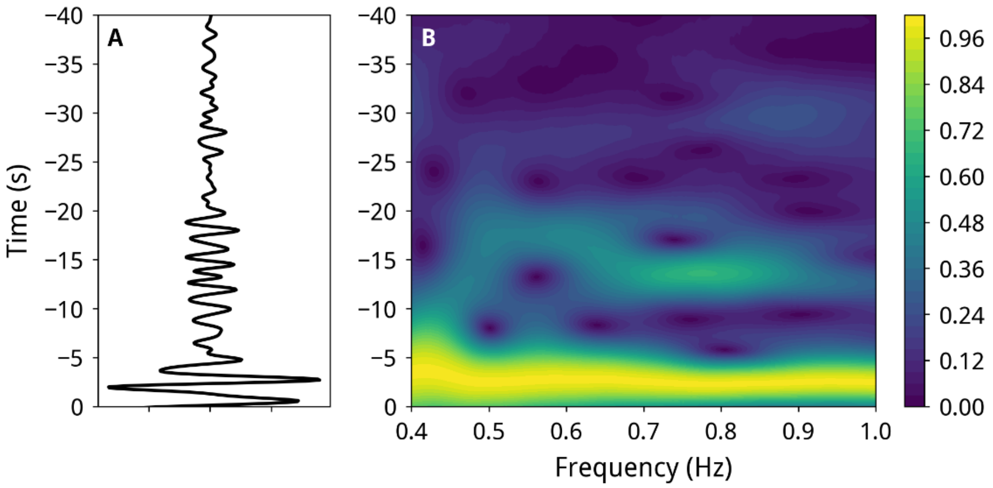

Fig. S8. Frequency content with lag time of an NCF at FLUR in the frequency band 0.4 to 1.0 Hz. (A) Acausal part of the NCF between the north and vertical components at station FLUR in the frequency band 0.4-1.0 Hz. (B) Frequency-time analysis (FTAN) of the NCF in A.

We also need to estimate the group velocity in this frequency range; we use 1.4 km/s and 1.2 km/s at frequencies of 1.0 Hz and 0.5 Hz, respectively, from a tomography study at a volcano in west Iceland (*55*). We can now estimate the radius of the Fresnel zone. By using the range of values of the variables v, t_0 and T we obtain a minimum estimate of 2.1 km and a maximum estimate of 4.8 km.

Unfortunately, phase velocity is more appropriate when measuring dv/v (from tiny phase changes in the coda); but picking phase velocity dispersion curves is more difficult. Nevertheless, this provides an order-of-magnitude estimate for the lateral sensitivity at station FLUR.
We can also estimate the wavelengths of Rayleigh waves. Using values of phase velocity estimated from the velocity model used in this study (1.9 km/s at 0.4 Hz and 1.4 km/s at 1.0 Hz) the wavelengths range from 1.4 to 4.8 km. This is similar to the estimate above, reinforcing our conclusion that the lateral sensitivity is on the scale of kilometers and is less than 5 km in this frequency band.

Section S4. Spatial variations in *dv/v*

We compare the time series of dv/v across the study area in fig. S9. The seasonal cycles described in the main text are generally consistent across the Northern Volcanic Zone, but there are notable exceptions at the stations in the south of the region around Vatnajökull ice cap and close to the coast. The environment may be quite different here, for example because of the effects of oceanic tidal loading and glacial meltwater.

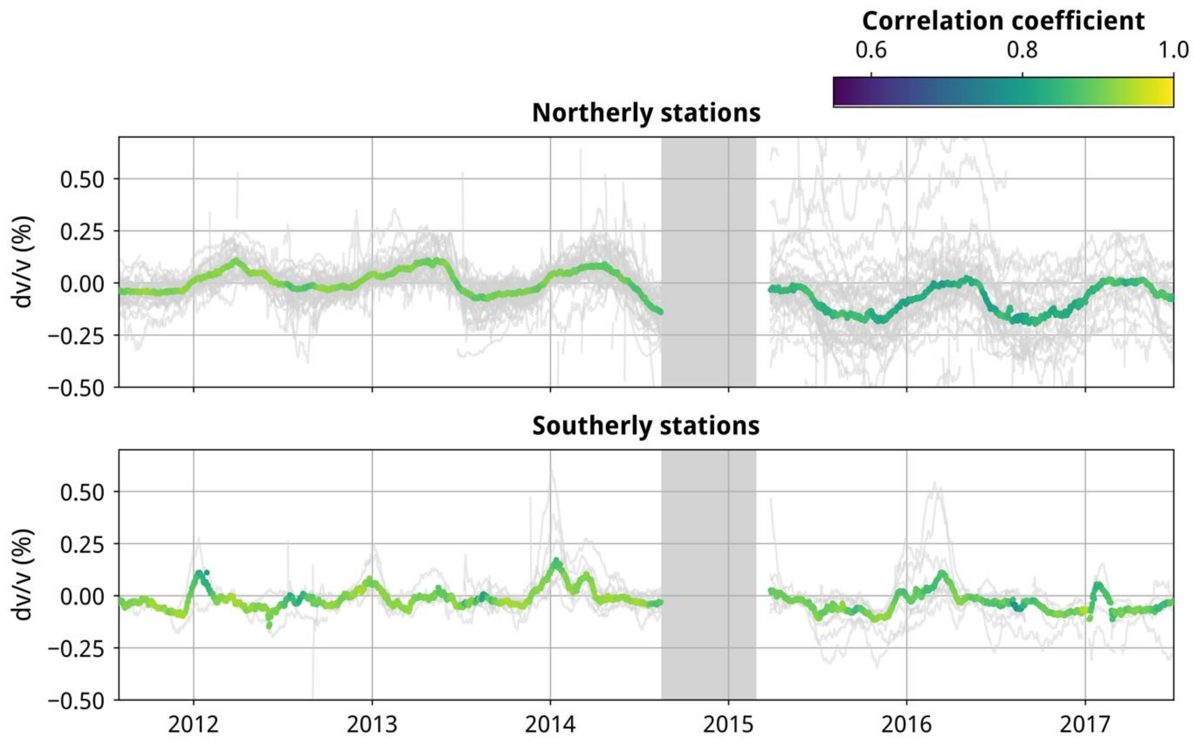

Fig. S9. Spatial variations in *dv/v* at 0.4 to 1.0 Hz. The stations are divided into those north and south of 64.4°N. dv/v measured from 30-days stacks in 0.4−1.0 Hz frequency band at individual stations (average of EZ, NZ, EN components). Averages shown, colored by average correlation coefficient with the reference functions.

Section S5. Comparison of measured and modeled GWL

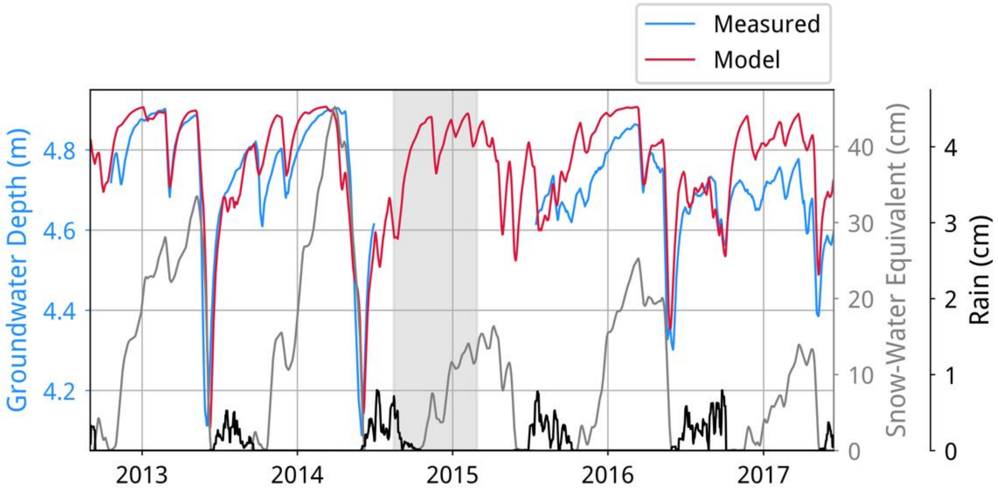

Fig. S10. Comparison of measured and modeled GWL. Snow thickness and rain data from IMO's meteorological model at the nearest point to borehole B5704, where GWL is measured. GWL modeled according to equation 2 with values of 0.24 for porosity, ϕ, and 0.06 for the decay constant, a.

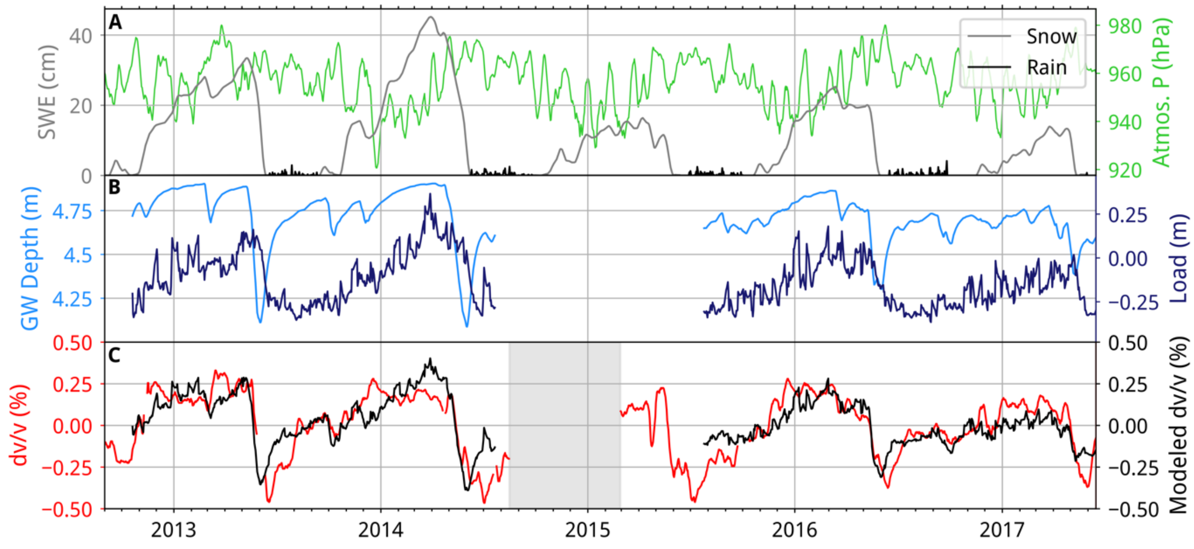

Fig. S11. Model of seasonal variations in *dv/v* at station SVA. (**A**) Weather from IMO's meteorological model at the nearest point to borehole B5704 (600 m south of station SVA, see Fig. 1). Snow thickness as snow-water equivalent shown in gray. Rainfall, at the same scale, in black. Atmospheric pressure in green. A 10-day rolling mean is applied to all weather data, to aid comparison with dv/v. (**B**) Groundwater depth below surface measured at borehole B5704 shown in light blue. Modeled total load (combining snow, atmospheric pressure and GWL, see text for details) shown in dark blue, at the same scale. (**C**) dv/v measured in 2−4 Hz band at station SVA (10-day stack, average of components NE, NZ and EZ) shown in red. Static references are used (a stack of data before 15 August 2014); moving references were tested and dv/v changes were of the same amplitude, but issues with drift (see text) mean that – in this case – static references are more helpful for comparison with weather data. Modeled dv/v in black, calculated by fitting dv/v using equation 5 with an OLS regression, as in Fig. 5.

Section S6. Forward model of changes in *dv/v* from Rayleigh wave phase velocities

In order to investigate further the effects of a changing surface load on dv/v, we have constructed a forward model of surface-wave velocity. We assume that adding a load at the surface is equivalent to shifting the velocity profile, as if everything were buried deeper than before, with the depth shift corresponding to the additional weight. The advantage of this simple approach is that we do not need to assume any values for the elastic parameters of the rock, which are poorly constrained (*56*). We calculate the phase-velocities of Rayleigh waves at different periods for the velocity profile shown in fig. S1. We then shift the velocity profile by 17 cm, which is the approximate thickness of basalt equivalent to the weight of 50 cm of water, and recalculate the phase velocities. In other words, when a load is added, the velocity is slightly faster at all depths below the seismometer.
Predicted values of dv/v are: 0.001 % at 0.1 Hz, 0.01 % at 0.4 Hz; 0.05 % at 1 Hz; 0.13 % at 2 Hz at 0.14 % at 4 Hz. The coefficients of load determined above are equivalent to 0.55 % and 0.04 % increases in dv/v for a surface load of 50 cm water-equivalent at 2–4 Hz and 0.1–0.4 Hz, respectively. These measured values are of the same order as those predicted for frequencies of at least 0.4 Hz, and the increasing sensitivity at higher frequencies agrees with our observations. This is encouraging, given the simplicity of the method and the large uncertainty in the shallow velocity structure, to which surface waves are highly sensitive (*51*). However, predicted values are consistently smaller than observed, which may be because non-linear effects are also at play, as has been suggested in other studies (*57*). As discussed in the Methods section, we use a combination of the regional 1D velocity model from (*21*) and a generic model for the shallow velocity structure at volcanoes (*52*), which is significantly slower. Interestingly, if we do not include these slower velocities in the top 500 m, the predicted values of dv/v are two orders-of-magnitude smaller. This indicates that the effects of elastic loading on relative seismic velocity are likely to be far more significant where slow, compressible material is present, such as in volcanic environments.

Section S7. Modeling pore pressure variations

We use groundwater level (GWL) as a proxy for pore-pressure when modeling the seasonal variations in dv/v because of the availability of borehole data, which we can use to calibrate our model. Chen & Talwani (*58*) and Talwani *et al.* (*36*) provide a detailed explanation of how pore-pressure varies for an input of water at the surface in rock containing narrow, fluid-filled fractures; the pore-pressure response is twofold. Firstly, due to the elastic load of the water input, the porous rock is compressed and − since fluids remain confined within it − the pore-pressure instantaneously increases. The second pore-pressure response is due to fluid diffusion (i.e. time delayed). Rivet et al. (*35*) and Wang et al. (*14*) use this second effect to model changes in dv/v at Piton de la Fournaise volcano and in Japan, respectively. Figure S12 shows pore-pressure variations through time averaged over 0−3 km depth, calculated following their methodology, but using our model of water input at the surface (including both snow-melt and rain). We tested values of 4.0 m^2/s and 1.0 m^2/s for the

hydraulic diffusivity, *c*, following Rivet *et al.* and Wang *et al.* respectively. Figure S12 shows that the time series of pore-pressure calculated using these two values are both very similar to the GWL time series. The advantage of using pore-pressure diffusion is that, in theory, varying behavior with depth can be studied. However, as explained in the main text, we have not attempted this due to (a) uncertainty about the value of hydraulic diffusivity, *c*, in this region, (b) because the permeability structure in central Iceland is likely to be layered (*39*), meaning a single value of *c* is likely to be inappropriate, and (c) because further work is needed to fully understand the depth sensitivity of the coda of NCFs. These results show that, when pore-pressure changes at shallow depths are included, the diffusive pore-pressure time series is very similar to that of GWL. Our seasonal model using GWL is therefore a reasonable first attempt at understanding how fluids affect dv/v in central Iceland.

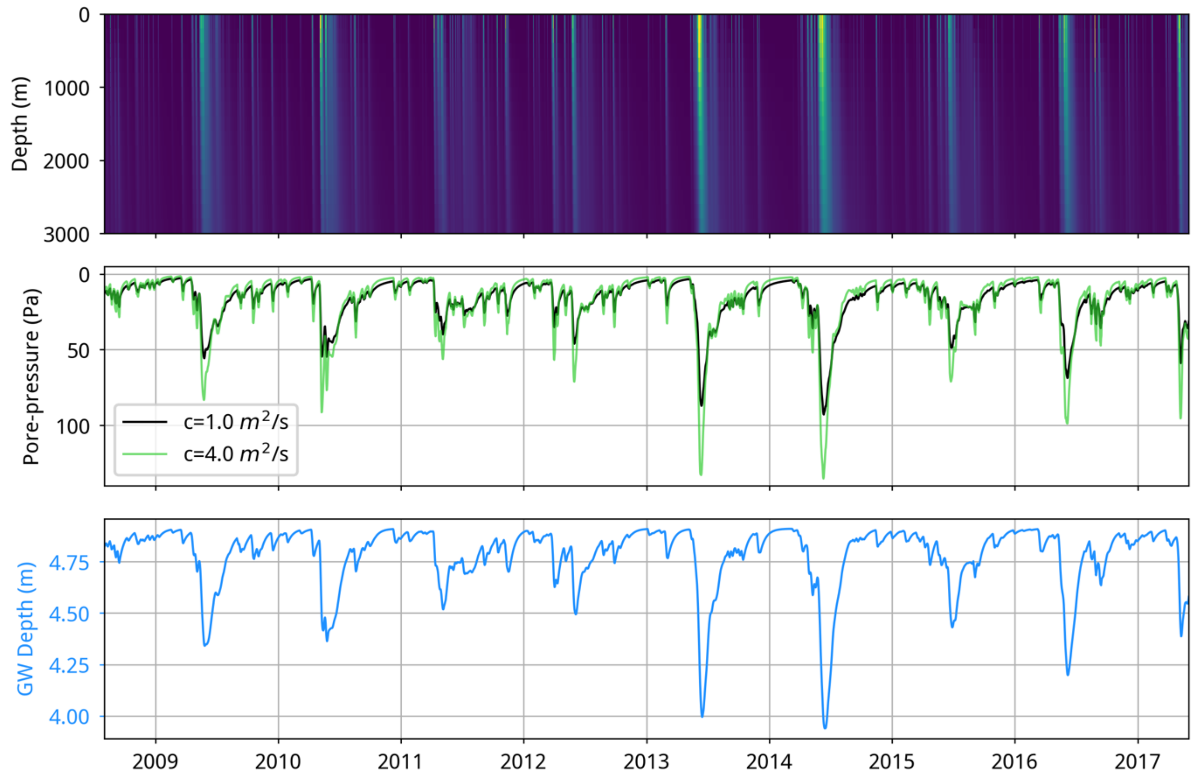

Fig. S12. Comparison of pore pressure and GWL models. (A) Pore-pressure with depth using a value for hydraulic diffusivity, c, of 4.0 m^2/s. Water input at the surface is as in Fig. 5: rainfall and snow-meltwater from IMO's meteorological model, averaged between stations KODA, FLUR, KRE and HELI. (B) Average pore-pressure in the top 3 km for c = 4.0 m^2/s (green) and c = 1.0 m^2/s (black). (C) The model of groundwater level shown in Fig. 5.

Section S8. Seasonal variation in *dv/v* and frost

As discussed in the main text, we have considered frost as a potential cause of the seasonal variations in dv/v. Figure S13 shows that there is an anti-correlation between dv/v and temperature, which is as

expected, given that dv/v is high when snow thickness is also high. We also show measurements of temperature made by the seismometers' internal thermistors. The differences between the independent measurements of temperature are likely to occur because of ground insulation and because the instrument generates some heat. Temperatures measured by the instruments themselves are useful because they are a more direct measurement of ground temperature. There is a steady ground temperature through the winter, then a rapid warming beginning in ~May or June. The frost may thaw later than the start of snow-melting because the snow itself insulates the ground to some extent. Crucially, the ground warming occurs later than the annual decreases in dv/v, which suggests that frost is unlikely to be the dominant mechanism.

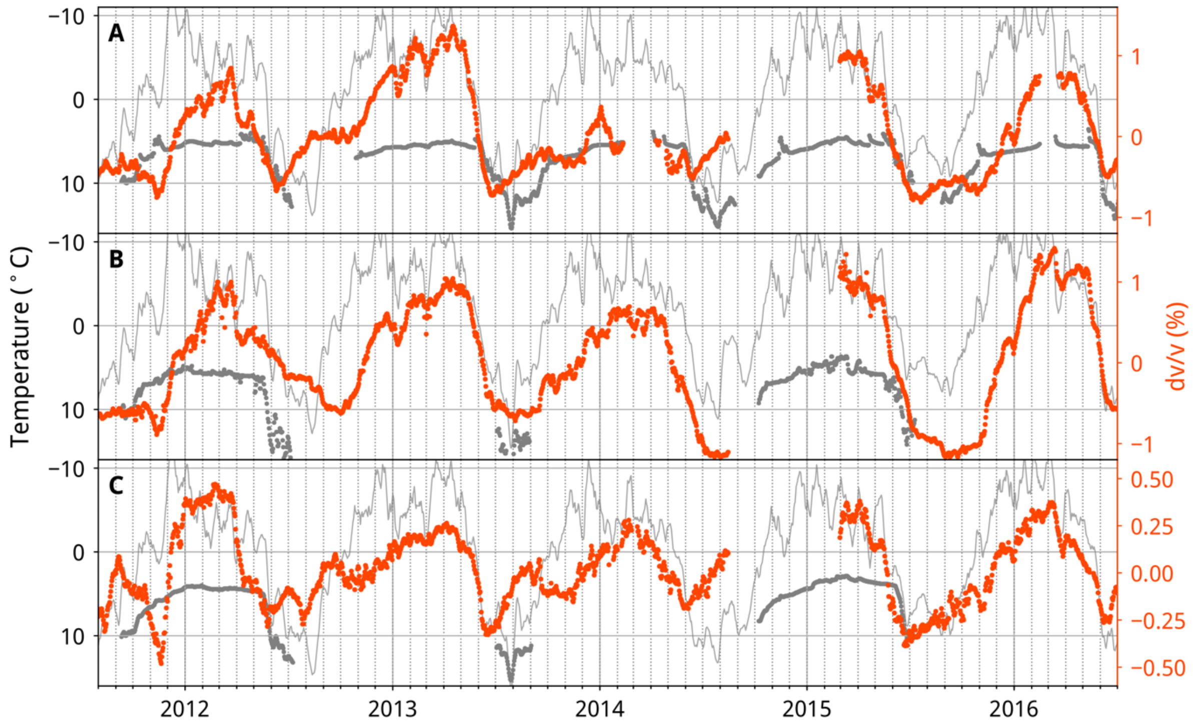

Fig. S13. Comparison of *dv/v* and temperature data. Temperature and dv/v at stations MIDF (**A**), LIND (**B**) and HELI (**C**). Light gray line is air temperature from IMO's meteorological model at the nearest point to the station. Dark gray circles show the temperature measured by the seismometers' internal thermistors. Orange circles are an average of dv/v measured from the three component pairs at the individual station, at 2–4 Hz.

Parameters used for measuring dv/v

Table S1. MSNoise parameters.

Frequency band (Hz)	MWCS window width (s)	MWCS window overlap (s)	dt/t window width (MWCS and stretching, s)	dt/t minimum lag for single-stations (MWCS and stretching, s)	Stretching range (%)	Stack length (days)	Reference style
0.1 – 0.4	20	5	50	24	-1 to 1	30	Static
0.4 – 1.0	8	2	30	20	-2 to 2	30	Static
1 – 2	4	1	15	8	-5 to 5	5	Moving
2 – 4	2	0.5	8	4	-5 to 5	5	Moving
4 – 16	0.5	0.125	2	1	-5 to 5	5	Moving

Parameter	Value
Clipping RMS amplitude for temporal normalization	3
Down-sampled rate	10 Hz
Down-sampled rate (4-16 Hz only)	50 Hz
Pre-process high-pass filter	0.01 Hz
Pre-process low-pass filter	4 Hz
Pre-process low-pass filter (4-16 Hz only)	24 Hz
Minimum coherence (MWCS)	0.6
Maximum dt (MWCS)	10 s
Maximum dt error (MWCS)	0.1
Maximum error in dt/t regression (MWCS)	0.1
Sides used in dt/t measurement	Both
Minimum correlation coefficient (stretching)	0.4
